# Dual Molecular Mechanisms Govern Escape at Immunodominant HLA A2-Restricted HIV Epitope

**DOI:** 10.3389/fimmu.2017.01503

**Published:** 2017-11-10

**Authors:** David K. Cole, Anna Fuller, Garry Dolton, Efthalia Zervoudi, Mateusz Legut, Kim Miles, Lori Blanchfield, Florian Madura, Christopher J. Holland, Anna M. Bulek, John S. Bridgeman, John J. Miles, Andrea J. A. Schauenburg, Konrad Beck, Brian D. Evavold, Pierre J. Rizkallah, Andrew K. Sewell

**Affiliations:** ^1^Cardiff University School of Medicine, University Hospital, Heath Park, Cardiff, United Kingdom; ^2^Department of Microbiology and Immunology, Emory University, Atlanta, GA, United States; ^3^James Cook University, Cairns, QLD, Australia; ^4^Cardiff University School of Dentistry, University Hospital, Heath Park, Cardiff, United Kingdom

**Keywords:** T-cell, T-cell receptor, HIV, immune escape, MHC

## Abstract

Serial accumulation of mutations to fixation in the SLYNTVATL (SL9) immunodominant, HIV p17 Gag-derived, HLA A2-restricted cytotoxic T lymphocyte epitope produce the SLFNTIAVL triple mutant “ultimate” escape variant. These mutations in solvent-exposed residues are believed to interfere with TCR recognition, although confirmation has awaited structural verification. Here, we solved a TCR co-complex structure with SL9 and the triple escape mutant to determine the mechanism of immune escape in this eminent system. We show that, in contrast to prevailing hypotheses, the main TCR contact residue is 4N and the dominant mechanism of escape is not *via* lack of TCR engagement. Instead, mutation of solvent-exposed residues in the peptide destabilise the peptide–HLA and reduce peptide density at the cell surface. These results highlight the extraordinary lengths that HIV employs to evade detection by high-affinity TCRs with a broad peptide-binding footprint and necessitate re-evaluation of this exemplar model of HIV TCR escape.

## Introduction

CD8^+^ cytotoxic T lymphocytes (CTLs) form a critical component in immune control of HIV infection. However, the virus has adopted numerous strategies to evade detection by these important immune cells (1, 2). Notably, the high rate and poor fidelity of replication produces a viral quasispecies to allow rapid selection of sequence variants that evade CTLs. This immune escape mutation is often associated with loss of viral fitness (3). The effects of immune escape from some HLA class I alleles, particularly HLAs B27 and B57 (3, 4), can be significant and delay progression to AIDS. There has been considerable work on immune escape from HIV epitopes presented by the most frequent HLA worldwide, HLA A2 (5). The majority of these studies have focussed on the immunodominant response through the p17 HIV Gag-derived epitope SL9 (amino acids 77–85, sequence SLYNTVATL) (6–23). Responses to SL9 are uncommon in acute infection but are dominant during the long, asymptomatic, chronic infection period where over 70% of HLA A2^+^ HIV-infected individuals mount a strong response to this epitope (13, 18, 19). Immune escape from SL9-specific CTL during this period has been studied extensively and is known to follow well-described patterns (6, 7). The leucine residues at positions 2 and 9 in SL9 are located within an α-helix (21) and are critical for trimerisation of the P17 Gag protein (24). Mutations within these residues are believed to produce highly compromised virus (11, 24). These residues also act as the principal anchors for securing SL9 into the HLA A2-binding groove (22) and ensure that the virus does not use the archetypical escape strategy of mutating primary MHC anchors to block epitope presentation at the infected cell surface (25–27). Instead, in the face of SL9-specific CTL pressure, HIV has been observed to undergo preferred mutation at positions 3, 6, and 8 in the epitope (6, 7, 9). The dominant mutations seen at these positions (Y3F, V6I, and T8V) correlate with a lower viral load suggesting that they result in a loss of viral fitness, a notion that is supported by the rapid reversion to the wild-type SL9 sequence in the absence of T-cell selection pressure (6, 7). The available evidence suggests that HIV balances the combined pressures of immune escape vs. viral fitness at the SL9 epitope by successive fixation of mutations at positions 3, 6, and 8 in the subtype A, B, and D SL9 SLYNTVATL consensus sequence over a >10-year period (6, 7). Indeed, McMichael and colleagues have described the triple mutant SL**F**NT**I**A**V**L sequence (3F6I8V; mutations in bold underlined text) as the “ultimate” escape mutant in SL9 (6).

Here we use structure and protein biophysics to deconstruct how HIV escapes from the well-studied (9–11) SL9-specific 868 TCR. We add to the existing data by providing the first ever TCR–pMHC structures in this exemplar model of how HIV evades engagement by host TCRs. We find that the 868 TCR interacts with HLA A2–SLYNTVATL (A2–peptide from here on in) with the expected binding topography but exhibits an unusual degree of structural rearrangement upon interaction with A2–SLYNTVATL. The 868 TCR also contacts all five central residues in SL9 (positions 3–8) and binds with an unprecedentedly high affinity for a natural TCR. Surprisingly, 4N is the major contact residue—not the 3Y, 6I, or 8T that mutate to precipitate immune escape. This enabled the generation of a high-resolution structure of the 868 TCR in complex with the SL**F**NT**I**A**V**L ultimate escape variant. These results challenge current thinking on the mechanism by which HIV escapes at SL9 and demonstrate the extraordinary tactics that the virus can employ to escape from CTL responses that can involve TCRs with a very broad footprint and a very high natural binding affinity.

## Materials and Methods

### Construct Design

The 868 TCR α and β chains, HLA A2 heavy chain, and β2m chain were generated by PCR mutagenesis and cloning. All sequences were confirmed by automated DNA sequencing (Lark Technologies). TCR expression constructs were designed with a disulphide linked construct to produce the soluble domains (variable and constant) for both the α (residues 1–207) and β chains (residues 1–247) (28, 29). The HLA A2 heavy chain (residues 1–248) (α1, α2, and α3 domains), tagged, or not tagged with a biotinylation sequence, and β2m (residues 1–100) were also cloned and used to make the pMHCI complexes. The TCR α and β chains, the HLA A2 α chain, and β2m sequences were inserted into separate pGMT7 expression plasmids under the control of the T7 promoter (30).

### Protein Expression, Refolding, and Purification

Competent Rosetta DE3 *E. coli* cells were used to produce the TCR α and β chains, HLA A2 heavy chain, and β2m in the form of inclusion bodies using 0.5 mM IPTG to induce expression and protein were chemically refolded as described previously (31).

#### pMHCI Biotinylation

Biotinylated pMHCI was prepared as previously described (32).

### Surface Plasmon Resonance (SPR) Experiments

Surface plasmon resonance equilibrium binding analysis was performed using a BIAcore T100™ equipped with a CM5 sensor chip as previously reported (32, 33). HLA DR1, generated as in Ref. (34), was used as a negative control on flow cell 1. SPR kinetic analyses were carried out to determine the *K*_D_ values for the TCR, at 25°C. For all kinetic experiments, approximately 300 RUs of pMHC was coupled to the CM5 sensor chip surface. The TCR was then injected at concentrations ranging from 10 times above and 10 times below the known *K*_D_ of the interaction at 45 µl/min. The *K*_D_ values were calculated assuming 1:1 Langmuir binding [AB = B × AB_MAX_/(*K*_D_ + B)], and the data were analysed using a global fit algorithm (BIAevaluation™ 3.1). The *k*_on_, *k*_off_, and *K*_D_ values were calculated by global fitting of the data using BIAevaluation™ 3.1 software. For the thermodynamics experiments we used the *K*_D_ determined by SPR at different temperatures with the standard thermodynamic equation Δ*G* = *RT* ln *K*_D_ and the standard non-linear van’t Hoff equation [Δ*G*° = Δ*H*°− *T*Δ*S*° + ΔCp°(*T*°− *T*_0_)°− *T*ΔCp° ln (*T*/*T*_0_)] with *T*_0_ = 298 K. For stability experiments, single injections of 50 µM 868 TCR over the SL9 variants were performed after different incubation times at 37°C, and the RU values were recorded.

### Isothermal Titration Calorimetry (ITC)

Isothermal titration calorimetry experiments were performed using a Microcal VP-ITC (GE Healthcare) as previously described (35), with 30 µM pHLA-I in the calorimeter cell and 210 µM soluble 868 TCR in the syringe. Buffer conditions were 20 mM Hepes (pH 7.4) containing 150 mM NaCl and 20 injections of 2 µl volume each were performed. Results were processed and integrated with the Origin 6.0™ software distributed with the instrument. ITC experiments were performed in duplicate.

### Adhesion Frequency Assay

We used an adhesion frequency assay to measure the 2D affinity of TCR–pMHC interactions at the cell membrane as previously described (36). Briefly, human T-cells transfected with the 868 TCR were mounted onto one micro-pipette and, on the other pipette, human red blood cells coated with pMHC by biotin–streptavidin coupling served as both a surrogate APC and an adhesion sensor for detecting the TCR–pMHC interaction. Site densities of TCR and pMHC were measured by flow cytometry as previously described (37). All assays were performed using at least 5 cell pairs, and calculated as an average of 100 cell–cell contacts.

### Crystallisation, Diffraction Data Collection, and Model Refinement

All protein crystals were grown at 18°C by vapour diffusion *via* the sitting drop technique. 200 nl of 1:1 molar ratio TCR and pMHCI (10 mg/ml) in crystallisation buffer (10 mM Tris pH 8.1 and 10 mM NaCl) was added to 200 nl of reservoir solution. 868 crystals were grown in TOPS (38) in 0.1 M sodium cacodylate pH 6.5, 20% PEG 8000, and 0.2 M ammonium sulphate (38). 868-A2–SLYNTVATL crystals were grown in TOPS in 0.1 M sodium cacodylate pH 6.0, 15% PEG 4000 and 0.2 M ammonium sulphate (38). 868-A2–SLYNT**I**ATL co-crystals were grown in TOPS in 0.1 M sodium cacodylate pH 6.0, 15% PEG 4000 and 0.2 M ammonium sulphate (38). 868-A2–SL**F**NT**I**A**V**L crystals were grown in TOPS1 in 0.1 M sodium cacodylate pH 5.5, 15% PEG 4000 and 0.2 M ammonium sulphate (38). A2–SLYNT**I**ATL crystals were grown in TOPS in 0.1 M sodium cacodylate pH 6.0, 25% PEG 4000 and 0.2 M ammonium sulphate (38). A2–SL**F**NT**I**A**V**L crystals were grown in TOPS in 0.1 M sodium cacodylate pH 6.0, 25% PEG 4000 and 0.2 M ammonium sulphate (38). All crystals were soaked in 30% ethylene glycol before cryo-cooling. All crystallisation screens and optimisation experiments were completed using an Art-Robbins Phoenix dispensing robot (Alpha Biotech Ltd, UK). Data were collected at 100 K at the Diamond Light Source, Oxfordshire. All datasets were collected at a wavelength of ~0.98Å using an ADSC Q315 CCD or PILATUS Pixel detectors. Reflection intensities were estimated with the XIA2 package (39) and the data were scaled, reduced and analysed with SCALA and the CCP4 package (40). Structures were solved with molecular replacement using PHASER (41). Sequences were adjusted with COOT (42) and the models refined with REFMAC5. Graphical representations were prepared with PYMOL (43). The reflection data and final model coordinates were deposited with the PDB database (868, PDB: 5NMD; 868-A2–SLYNTVATL, PDB: 5NME; 868-A2–SLYNT**I**ATL, PDB: 5NMF; 868-A2–SL**F**NT**I**A**V**L, PDB: 5NMG; A2–SLYNT**I**ATL, PDB: 5NMH; A2–SL**F**NT**I**A**V**L, PDB: 5NMK).

### Thermal Stability CD Analysis of HLA A2 Complexes

Thermal stability of HLA A2/β2m/peptide complexes was determined by circular dichroism spectroscopy following the change of ellipticities Θ at 218 nm using an Aviv 215 instrument (Aviv Biomedical Inc., Lakewood, NJ, USA). Proteins were dissolved in 137 mM NaCl, 3 mM KCl, 8 mM Na_2_HPO_4_, and 1 mM KH_2_PO_4_, pH 7.4, at concentrations of ~3 μM as determined spectroscopically using calculated extinction coefficients (44). Melting curves were recorded from 4°C up to a maximum temperature when protein aggregation was observed using a heating rate of ~0.5°C/min. Melting curves were analysed assuming a two-state trimer-to-monomer transition from the native (N) to unfolded (U) conformation N_3_ ↔ 3U with an equilibrium constant *K* = [U]^3^/[N_3_] = *F*/[3*c*^2^ (1 − *F*)^3^], where *F* and *c* are the degree of folding and protein concentration, respectively. Data were fitted as described (45) using the non-linear least-squares routine of Origin V7.5 (OriginLab, Northampton, MA, USA). Fitted parameters were the melting temperature Tm, van’t Hoff’s enthalpy Δ*H*_vH_ at the transition midpoint, and the slope and intercept of the native baseline assumed as a linear function of the temperature. As protein complexes aggregated at various degrees of unfolding, the ellipticity of the unfolded state was set as a constant of −4,500 deg cm^2^ dmol^−1^ (46).

### Generation of 868 TCR Transgenic T-Cells

868 TCR construct (codon-optimised for human expression) was cloned into the third-generation lentiviral expression vector pELNS (kindly provided by James R. Riley, University of Pennsylvania, PA, USA). pELNS vector was engineered to contain rat CD2 (rCD2) gene to be used as a marker of lentiviral transduction of target cells. 868 TCR-α and TCR-β, and rCD2 were separated by a pair of “self-cleaving” 2A sequences (11). Lentiviral particles were prepared by calcium phosphate transfection of packaging plasmids (pRSV-Rev, pMDLg/pRRE, pMD2.G) together with 868-encoding pELNS into HEK293T cells. Supernatant was harvested 48- and 72-h post transfection and was subsequently concentrated by ultracentrifugation. Primary CD8^+^ T-cells were obtained from healthy donor blood bags (Welsh Blood Service) by density gradient centrifugation and positive selection using CD8 MicroBeads (Miltenyi Biotec, Bergisch Gladbach, Germany). Isolated T-cells were stimulated overnight with Gibco human T-activator CD3/CD28 Dynabeads (ThermoFisher Scientific, Waltham, MA, USA) at 3:1 bead to T-cell ratio, and transduced with concentrated lentiviral stock in presence of 5 µg/ml polybrene (Santa Cruz Biotechnology, Santa Cruz, CA, USA). After 72 h rCD2^+^ cells were enriched by magnetic bead selection to yield >95% purity. For tetramer staining experiments, CD8^+^ T-cells were used without magnetic enrichment to leave a non-transduced internal control cell population. Similar methodology was used to transduce TCRβ chain negative Jurkat cells to allow the examination of tetramer binding in the absence of the CD8 glycoprotein.

### Tissue Culture

T2 cells were maintained as suspension cells in R10 (RPMI 1640 media, 10% FBS, penicillin, streptomycin, and l-glutamine, all Life Technologies, Paisley, UK) and passaged weekly or when required. Post enrichment, primary CD8^+^ T-cells were routinely expanded with allogeneic feeder PBMCs and phytohaemagluttinin (PHA) in T-cell media as previously described (47). 868 TCR expression was checked prior to assay using anti-rCD2 antibody and A2-SLYNTVATL tetramer, with the protein kinase inhibitor Dasatinib (48) (Axon Medchem, Reston, VA, USA), and unconjugated anti-PE antibody (PE001, BioLegend, London, UK) used to enhance tetramer staining, as previously described (47, 49).

### T-Cell Activation Assays

For peptide pulsing assays, T2 cells were incubated with peptide for 1 h in 0.5 ml of AIM-V serum free media (ThermoFisher Scientific) in 5 ml FACS tubes at 37°C and 5% CO_2_, followed by 4 washes with 4 ml of RPMI 1640 media. In assays using primary CD4^+^ T-cells as targets, these cells were purified from PBMC using magnetic microbead separation (Miltenyi Biotech). Peptides were synthesised to >95% purity, stored as 20 mM DMSO stocks at −80°C and 1 mM RPMI dilutions made on the day of assay. DMSO controls were treated the same as peptide pulsed. Post pulsing, T2 cells or CD4^+^ T-cells were incubated in 4.5 ml of AIM-V media at 37°C and 5% CO_2_ for 3–24 h or used immediately (0 h), such that cells were ready at the same time. 868 TCR transduced and non-transduced T-cells (30,000) were rested overnight in R5 (as for R10 but with 5% fetal bovine serum) then co-incubated in 96 U-well plates with T2 or CD4^+^ T-cells (60,000) in the presence of GolgiStop and GolgiPlug according the manufacturers (BD Biosciences, Oxford, UK), and anti-CD107a PE Antibody (H483, BD Biosciences). Controls included T-cells alone and T-cells with unpulsed T2 cells (negative control) or PHA. After 3.5–5 h of incubation, cells were labelled with the violet LIVE/DEAD fixable dead cell stain Vivid, anti-CD8 APC (BW135/80; Miltenyi Biotech) and anti-CD19 pacific-blue (T2 cells) (BioLegend), and rCD2 (CD4 assay) and anti-CD4 APC-Vio770 (M-T466, Miltenyi Biotech; CD4 assay) antibodies. Cells were then stained with anti-TNFα PE-Vio770 antibody (CA2, Miltenyi Biotec) using a Cytofix/Cytoperm kit (BD Biosciences) according to manufacturer’s instructions. Flow cytometry was performed on a FACSCanto II (BD Biosciences) and data analysed with FlowJo software (TreeStar, Ashland, OR, USA). For peptide alanine scans, 868 TCR transduced T-cells were rested overnight in R5 then incubated with T2 cells (60,000) and peptides at specified concentrations for 18 h in 96U-well plates in 100 µl of R5. Supernatants (50 µl) were used for MIP-1β ELISA (R&D Systems, Minneapolis, MN, Canada) and GraphPad Prism (GraphPad Software, Inc., La Jolla, CA, USA) used to fit non-linear regression curves and calculate EC_50_ for each peptide.

### T2 Peptide–HLA-A2 Stability Assays

For conventional peptide-binding assays, T2 cells were pulsed for 22 h with 10^−5^ M peptide in 0.2 ml of AIM-V media (ThermoFisher Scientific) in 5 ml FACS tubes at 37°C and 5% CO_2_, washed then stained with anti-HLA-A2 antibody (BB7.2 BioRad, Hercules, CA, USA) for 10 min at RT, washed with PBS, and fixed with 2% paraformaldehyde for 20 min on ice. Peptides were synthesised to >95% purity, stored as 20 mM DMSO stocks at −80°C, and 1 mM AIM-V dilutions made on the day of assay. DMSO controls were treated the same as peptide pulsed. For peptide on-rates, T2 cells were treated as before but for the durations specified in the results (1–3 h) then stained and fixed. For peptide off-rates, T2 cells were pulsed overnight, then a proportion of them stained immediately, and fixed as above, with remaining cells being washed in an excess of media (two times using 4 ml) and incubated in 4.5 ml of AIM-V for the specified times (h) at 37°C and 5% CO_2_, to allow peptide to dissociate, then stained and fixed as before. Flow cytometry was performed on a FACSCanto II (BD Biosciences) and data analysed with FlowJo software (TreeStar).

### Peptides Used in This Study

This study made use of the HIV SL9 peptide and variants thereof. The sequences and origin of these peptides, and other control peptides utilised, are listed in Table 1.

**Table 1:** Peptides used in this study.

Peptide sequence	Abbreviation	HLA-restriction	Parent protein
SLYNTVATL	SL9	HLA A*0201	HIV p17 Gag
SL**F**NTVATL	3F	HLA A*0201	HIV p17 Gag
SLYN**I**VATL	6I	HLA A*0201	HIV p17 Gag
SLYNTVA**V**L	8V	HLA A*0201	HIV p17 Gag
SL**F**N**I**VA**V**L	3F6I8V	HLA A*0201	HIV p17 Gag
GILGFVFTL	A2-flu	HLA A*0201	Influenza matrix
HPVGEADYFEY		HLA B*3501	EBV EBNA-1

## Results

### 868 TCR Binding to A2–SLYNTVATL Involves Extensive Structural Reorganisation

**Figure 1:**
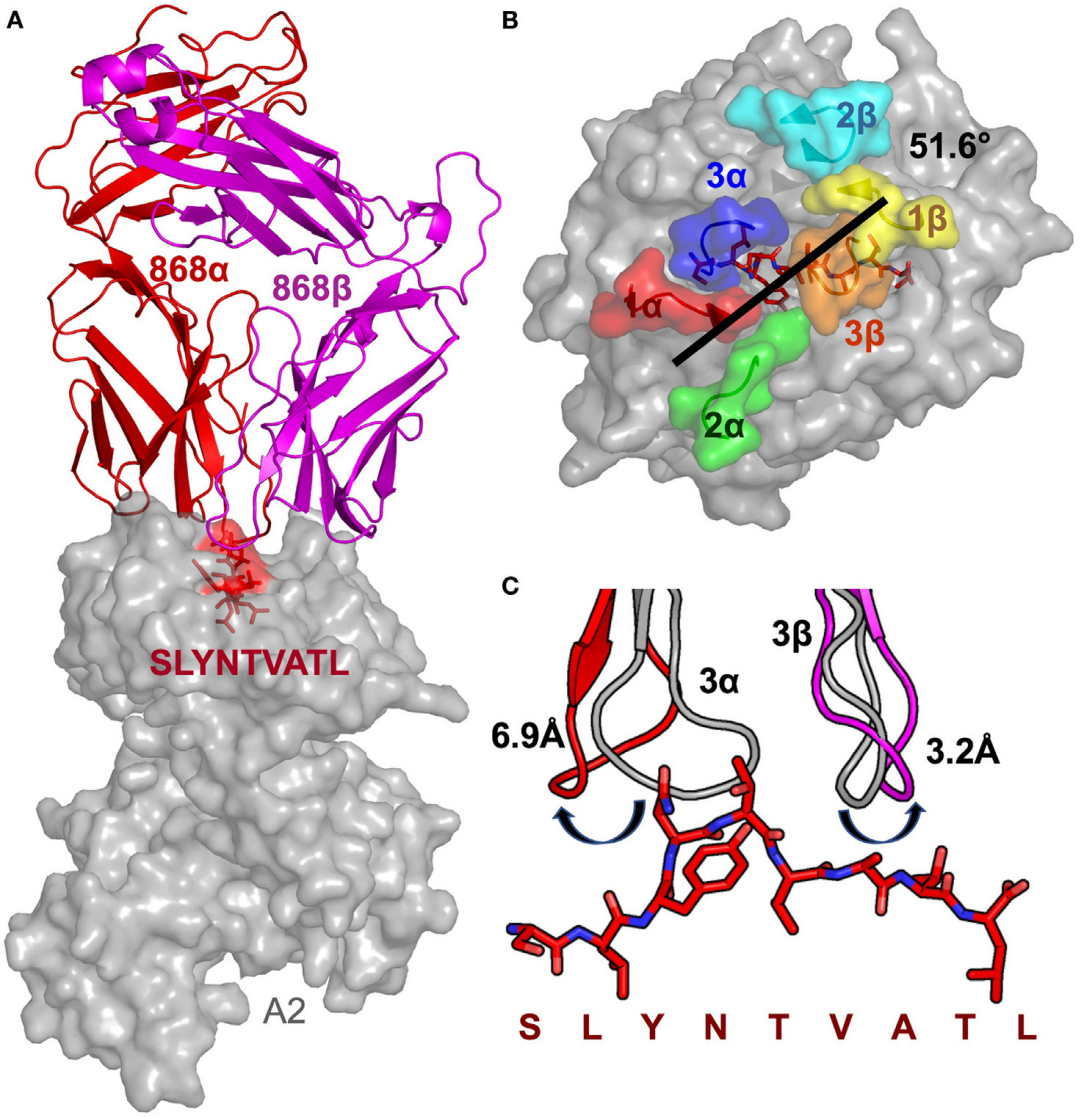
Structural analysis of the 868-A2–SL9-binding mode. **(A)** The overall binding mode of the 868 TCR (red and magenta cartoon) interacting with HLA A2 (grey surface) and the SLYNTVATL peptide (red sticks and surface). **(B)** Surface representation of the A2–SLYNTVATL complex looking down at the peptide. Positions of the 868 TCR CDR loops (1α—red, 2α—green, 3α—blue, 1β—yellow, 2β—cyan, 3β—orange) and the crossing angle of the TCR with the MHC groove is shown. **(C)** The positions of the 868 TCR CDR3 loops differ considerably between the ligated (red cartoon) and unligated (grey cartoon) structures.

Previous studies have demonstrated that over 1.7% of CD8^+^ T-cells from HLA A2^+^ HIV-infected patient 868, stained with A2–SLYNTVATL tetramer (10). 75% of these tetramer^+^ cells expressed a TRBV5-6^+^ TCR and an identical TRBV5-6 β-chain was observed in 10/18 transcripts sequenced in this study (CDR3 sequence CASSLSAVQNNEQF) (10). This same β-chain was later shown to be present in all A2–SLYNTVATL tetramer^+^ cells in a T-cell line grown from patient 868 in 1996 (9). TCR chain antibody staining showed that all of these A2–SLYNTVATL tetramer^+^ T-cells co-expressed a TRAV12-2 chain (11). Our previous study confirmed that CD8^+^ T-cells expressing this TCR were unable to control HIV virus *in vitro* (11). Nevertheless, we were able to use phage display to select an artificially enhanced version of the 868 TCR that could recognise the SL**F**NT**I**A**V**L ultimate escape variant; thereby engineering foresight into an immune receptor (11). The detection of common SL9 escape mutants on the surface of HIV-infected HLA A2^+^ cells by CD8^+^ T-cells expressing engineered, but not wild-type, 868 TCR reinforced earlier studies suggesting that these mutants are presented by HLA A2 and that escape from the wild-type epitope is mediated by loss of TCR binding (so-called “TCR escape”) (6, 7, 11, 18). To understand how positions 3, 6 and 8 in SL9 impinge on TCR binding, we solved the structure of the wild-type 868 TCR in complex with A2–SLYNTVATL to 2.9 Å resolution (Table 2). The 868 TCR bound to A2–SLYNTVATL with a conventional, centrally-located, diagonal orientation with the TCR α-chain positioned over the α2 helix of MHC class I and the TCR β-chain positioned over the α1 helix (Figure 1A). The TCR crossing angle of 51.6° lies within the normal range for human TCR–pMHC complexes (50), enabling positioning of the TCR CDR3 loops centrally over the peptide, and the CDR1 and CDR2 loops predominantly over the MHC helices (Figure 1B). To gain extra insight into the mechanism by which the 868 TCR adopted this binding mode, we also solved the structure of the unbound 868 TCR (Table 2). Comparison with the 868-A2–SLYNTVATL complex structure showed that extensive structural reorganisation of the CDR3 loops was required to avoid clashes with the solvent-exposed residues in the SL9 peptide. This resulted in an induced fit, or conformational pre-equilibrium binding mode where the TCR exists in two conformations in the unbound state, with the CDR3α loop “moving” 6.9 Å and the CDR3β moving 3.2 Å between the bound and unbound TCR structures (Figure 1C).

**Table 2:** Data collection and refinement statistics for unligated 868 TCR and complex structures.

	868 TCR	868-A2–SLYNTVATL	868-A2–SLYNTIATL	868-A2–SLFNTIAVL	A2–SLYNTIATL	A2–SLFNTIAVL
**Data collection**						
Space group	P 1 21 1	P 21 21 2	P 21 21 2	P 21 21 2	P 1 21 1	P 1 21 1
**Cell dimensions**						
*a, b, c* (Å)	87.5, 50.6, 114.3	211.1, 85.1, 113.2	207.6, 84.7, 112.1	209.36, 85.11, 113.15	56.1, 80.1, 57.6	55.84, 79.03, 58.37
α, β, γ (°)	90, 90.2, 90	90, 90, 90	90, 90, 90	90, 90, 90	90, 115.8, 90	90, 116.11, 90
Resolution (Å)	43.8–2.1 (2.13–2.07)	64.8–2.9 (3.01–2.94)	34.0–2.9 (2.96–2.89)	99.5–2.8 (2.82–2.75)	31.7–1.6 (1.59–1.55)	48.4–1.7 (1.70–1.66)
Beam line	I24	I03	I03	I04-1	I03	I24
Beam time code	6232-9	4532-1	6232-1	6232-10	4532-1	3262-9
*R*_merge_ (%)	5.0 (72.3)	12.5 (95.9)	15.1 (84.0)	11.7 (86.7)	6.6 (73.3)	7.4 (130.3)
*I/*σ*I*	12.1 (2.3)	12.4 (2.1)	10.9 (2.9)	13.0 (2.5)	12.2 (2.4)	9.5 (2.1)
Completeness (%)	98.6 (99.6)	99.1 (99.8)	100 (100)	99.9 (100)	99.4 (97.7)	98.9 (99.8)
Redundancy	3.5 (3.8)	7.5 (7.1)	7.9 (8.2)	7.4 (7.6)	4.1 (4.0)	3.6 (3.7)
No. reflections	60,507 (4,492)	44,013 (3,213)	45,223 (3,284)	53,560 (3,895)	66,024 (4,752)	53,691 (4,000)
**Refinement**						
No *R*_free_ reflections	3,051	2,212	2,223	2,717	3,346	2,724
*R*_work_/*R*_free_	18.9/22.3	19.4/28.9	18.8/27.5	19.2/27.0	14.4/19.4	17.5/20.8
**R.m.s. deviations**						
Bond lengths (Å)	0.0189	0.015	0.012	0.017	0.025	0.017
Bond angles (°)	1.970	1.665	1.483	1.954	2.107	1.832
Mean *B* value (Å^2^)	57.0	69.0	53.7	31.2	16.8	24.3
Wilson *B*-fac (Å^2^)	40.7	72.2	60.3	58.9	17.2	22.5
ML estimated coordinate error (Å)	0.144	0.387	0.365	0.300	0.049	0.070

*One crystal was used for solving each structure*.

*Figures in brackets refer to the highest resolution bin*.

**Figure 2:**
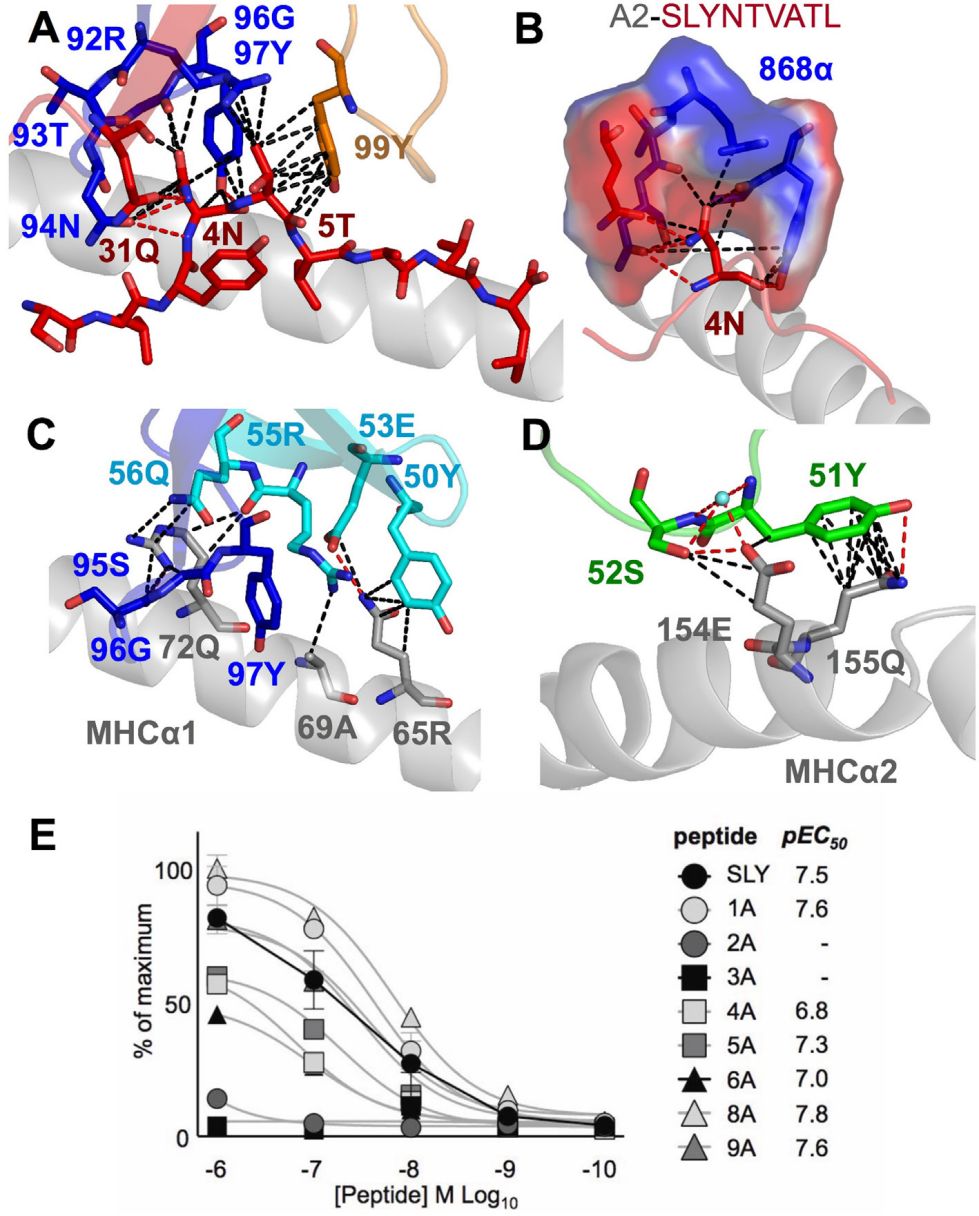
The 868 TCR focuses on residues 4N and 5T in the peptide. Hydrogen bonds show as red dotted lines, van der Waal’s contacts shown as black dotted lines in panels **(A–D)**. **(A)** The 868 TCR (CDR3α in blue, CDR1α red and CDR3β orange sticks) binding to the solvent-exposed peptide residues 4N and 5T in the peptide (red sticks). **(B)** The 868 TCR (CDR3α in blue, CDR1α red sticks and surface) forms a tight pocket around peptide residue 4N (red sticks) during binding. **(C,D)** The restriction triad residues 72Q, 69A, and 155Q (grey sticks) play a central role in 868 TCR–MHC-mediated interactions (TCR CDR3α residues shown in blue, CDR2β in cyan, and CDR1α in green sticks). **(E)** 868 TCR transduced CD8^+^ T-cells from two HLA A2^+^ donors were co-incubated with T2 cells and alanine-substituted peptides of SLYNTVATL (alanine already at position 7) overnight and supernatants used for MIP-1β ELISA. Non-linear regression curves and pEC_50_ for each peptide are displayed.

Closer examination of the interface between the TCR and the pMHC showed that the CDR3 loops almost exclusively made contacts with the peptide, forming a pincer around the solvent-exposed 4N and 5T residues in the peptide (Figures 2A,B). The TCR–peptide interaction included 10 hydrogen bonds and 52 vdW contacts, mainly focussed on 4N. The CDR1 and CDR2 loops primarily contacted the α helices of the MHC, including interactions with all three restriction triad residues (MHC residues 65R, 69A, and 155Q) as well as strong interactions with MHC residues 72Q and 154E (Figures 2C,D). Although most of the TCR–pMHC interactions were focussed on these 2 peptide residues and 5 MHC residues, the 868 TCR bound with a broad footprint, making additional bonds with all but 2 peptide residues (1S and 9L) and a further 14 MHC residues. In total, the 868 TCR made 20 hydrogen bonds, and 121 van der Waals (vdWs) contacts with A2–SLYNTVATL (Table 3), which is in the higher range compared to previously published viral TCR–pMHC complexes (35). In summary, the 868 TCR utilised an induced fit, or conformation selection, binding mode to form a pincer-like interaction around the central solvent-exposed portion of the peptide. Additional contacts with distal peptide residues enabled a relatively extensive and broad binding interface between the TCR and pMHC.

**Table 3:** Summary of co-complex structures of 868-A2–SLYNTVATL, 868-A2–SLYNTIATL, and 868-A2–SLFNTIAVL.

	868-A2–SLYNTVATL	868-A2–SLYNTIATL	868-A2–SLFNTIAVL
H-bonds (≤3.4 Å)	20	20	18
vdWs (≤4 Å)	121	118	128
Total contacts	141	138	146
CDR1/CDR2/CDR3 contacts (≤4 Å)			
α Chain	13/33/41	9/23/46	8/22/47
β Chain	0/17/37	0/27/43	0/24/45
Peptide contacts	62	56	63
MHC contacts	79	82	83
Crossing angle (°)	51.6	50.3	51.7
Buried surface area (Å^2^)	2,396.2	2,469	2,698
Surface complementarity			
TCR–MHC	0.50	0.51	0.55
TCR–peptide	0.72	0.70	0.71
TCR–pMHC	0.56	0.55	0.59

*HB, hydrogen bond; SB, salt bridge; WB, water bridge; vdWs, van der Waals interactions*.

*A 3.4 Å cut-off was used for HBs, SBs, and WBs, and a 4 Å cut-off was used for vdWs*.

### TCR Structure with the Ultimate SL9 Escape Variant

**Figure 3:**
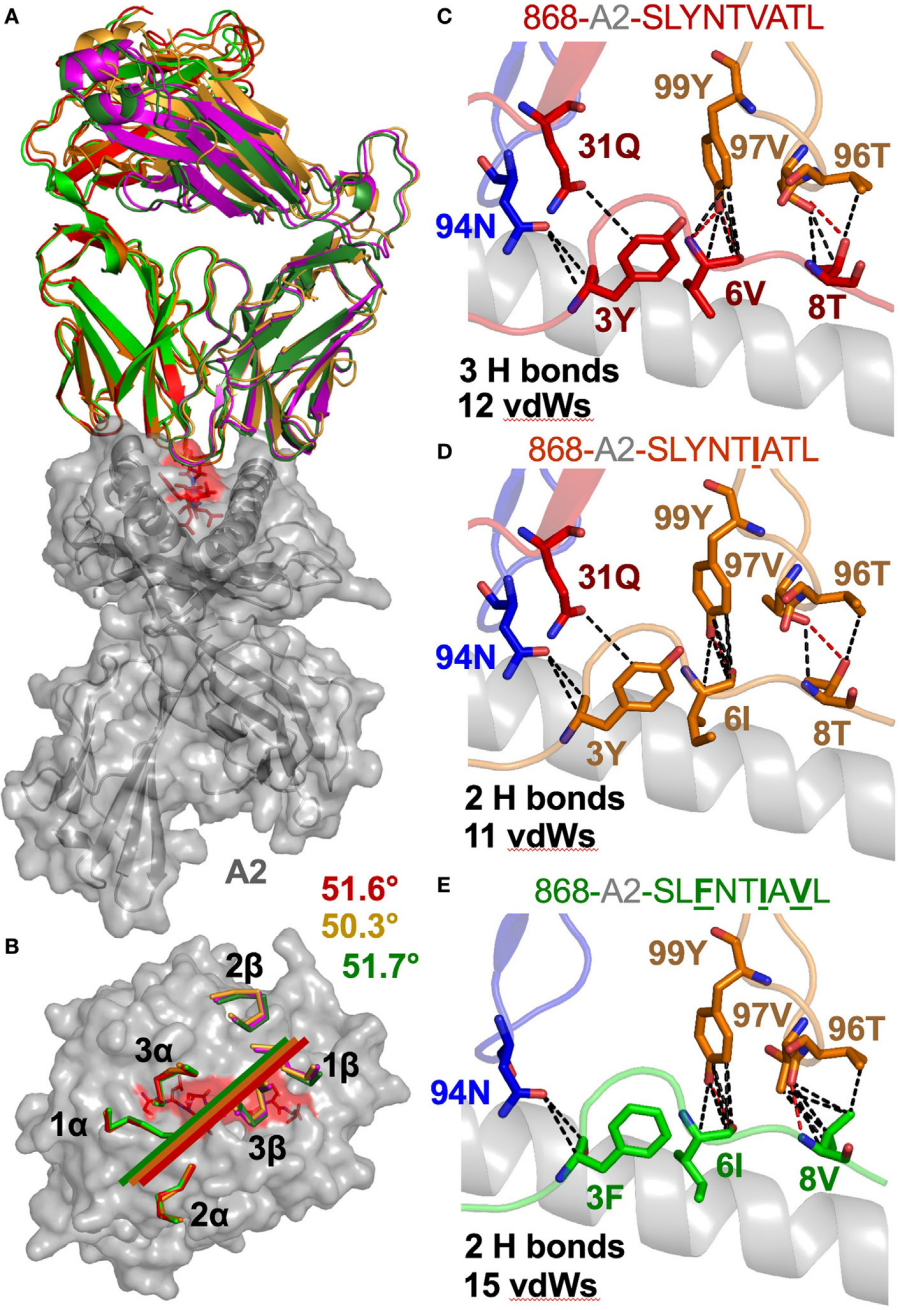
The 868 TCR uses a virtually identical conformation when interacting with the common escape mutants SLYNT**I**ATL and SL**F**NT**I**A**V**L. **(A)** Identical overall binding mode of 868 TCR interacting with A2–SLYNTVATL (red), A2–SLYNT**I**ATL (orange), and A2–SL**F**NT**I**A**V**L (green). HLA A2 in grey cartoon. **(B)** Surface representation of the A2–SLYNTVATL complex looking down at the peptide. Positions of the 868 TCR CDR loops in the A2–SLYNTVATL (red), A2–SLYNT**I**ATL (orange), and A2–SL**F**NT**I**A**V**L (green) complexes. Crossing angle of the TCR is indicated. **(C)** Contacts between the 868 TCR and residues 3Y, 6V, and 8T in the SLYNTVATL peptide that constitute the positions that a commonly mutated by HIV. **(D)** Contacts between the 868 TCR and residues 3Y, 6I, and 8T in the SLYNT**I**ATL peptide. Although slightly different, the total number of contacts between the 868 TCR and A2–SLYNT**I**ATL is similar to the 868-A2–SLYNTVATL complex. **(E)** Contacts between the 868 TCR and residues 3F, 6I, and 8V in the A2–SL**F**NT**I**A**V**L triple escape mutant peptide. Again, the total number of contacts between the 868 TCR and A2–SL**F**NT**I**A**V**L is similar to the complex with the wild-type index peptide. Hydrogen bonds shown as red dotted lines and van der Waal’s contacts shown as black dotted lines in panels **(C–E)**.

The 868-A2–SLYNTVATL complex structure demonstrated that the dominant contacts with the SL9 peptide were with the 4N residue rather than with the 3Y, 6V, or 8T residues that result in immune escape when substituted to create the SL**F**NT**I**A**V**L ultimate escape variant (6, 7). Alanine scan mutation across the SL9 peptide backbone showed an almost complete loss of recognition with the L2A and Y3A changes. Alanine substitution at positions 4, 5, and 6 had the next greatest effect whereas peptides substituted at positions 1, 8, and 9 exhibited recognition equivalent to the wild-type peptide (Figure 2E). In order to gain insight as to how natural escape mutations impinge on TCR binding, we also solved the structure of the 868 TCR with the SLYNT**I**ATL variant present in the patient at the time the blood sample was used to grow the 868T-cell clone (9), and the SL**F**NT**I**A**V**L ultimate escape variant (Table 2). Comparison of these structures with the 868 TCR in complex with the A2–SLYNTVATL index sequence showed that the 868 TCR engaged all the antigens in a similar conformation and with a similar interaction network (Figures 3A–E). We were initially surprised at being able to solve the structure of the 868 TCR in complex with the SL**F**NT**I**A**V**L peptide as this triple mutant is known to escape from CD8^+^ T-cells (6, 7). Indeed, this mutation is not well recognised by T-cells bearing the 868 TCR (9, 11). The failure of CD8^+^ T-cells to recognise the SL**F**NT**I**A**V**L mutation has previously been attributed to reduced interactions with the TCR (6, 7, 18, 23). Earlier structural analyses of A2–SLYNTVATL and A2–SL**F**NT**I**A**V**L have concluded that all three substitutions directly affected TCR recognition (6, 7). However, we observed only minor differences at the TCR–peptide interface with these two ligands. For example, the 868 TCR made 3 hydrogen bonds and 12 vdWs with peptide residues 3Y, 6V, and 8T in the SLYNTVATL peptide compared to 2 hydrogen bonds and 15 vdWs contacts with 3F, 6I, and 8V in the SL**F**NT**I**A**V**L peptide (Figures 3C,E). The total number of contacts in each complex (868-A2–SLYNTVATL: 141, 868-A2–SL**F**NT**I**A**V**L: 146) was also similar, demonstrating that the 868 TCR could tolerate mutations at residues 3, 6, and 8 in SL9 (Table 3). Thus, because our structural data were inconsistent with the hypothesis that SL**F**NT**I**A**V**L primarily affects TCR binding, and because sum contact numbers do not necessarily represent the accurate binding energy between two molecules, we undertook further structural and biophysical experimentation to determine the mechanism that results in poor T-cell recognition of SL**F**NT**I**A**V**L.

### A2–SLYNTVATL Does Not Alter Conformation When Bound to 868 TCR

**Figure 4:**
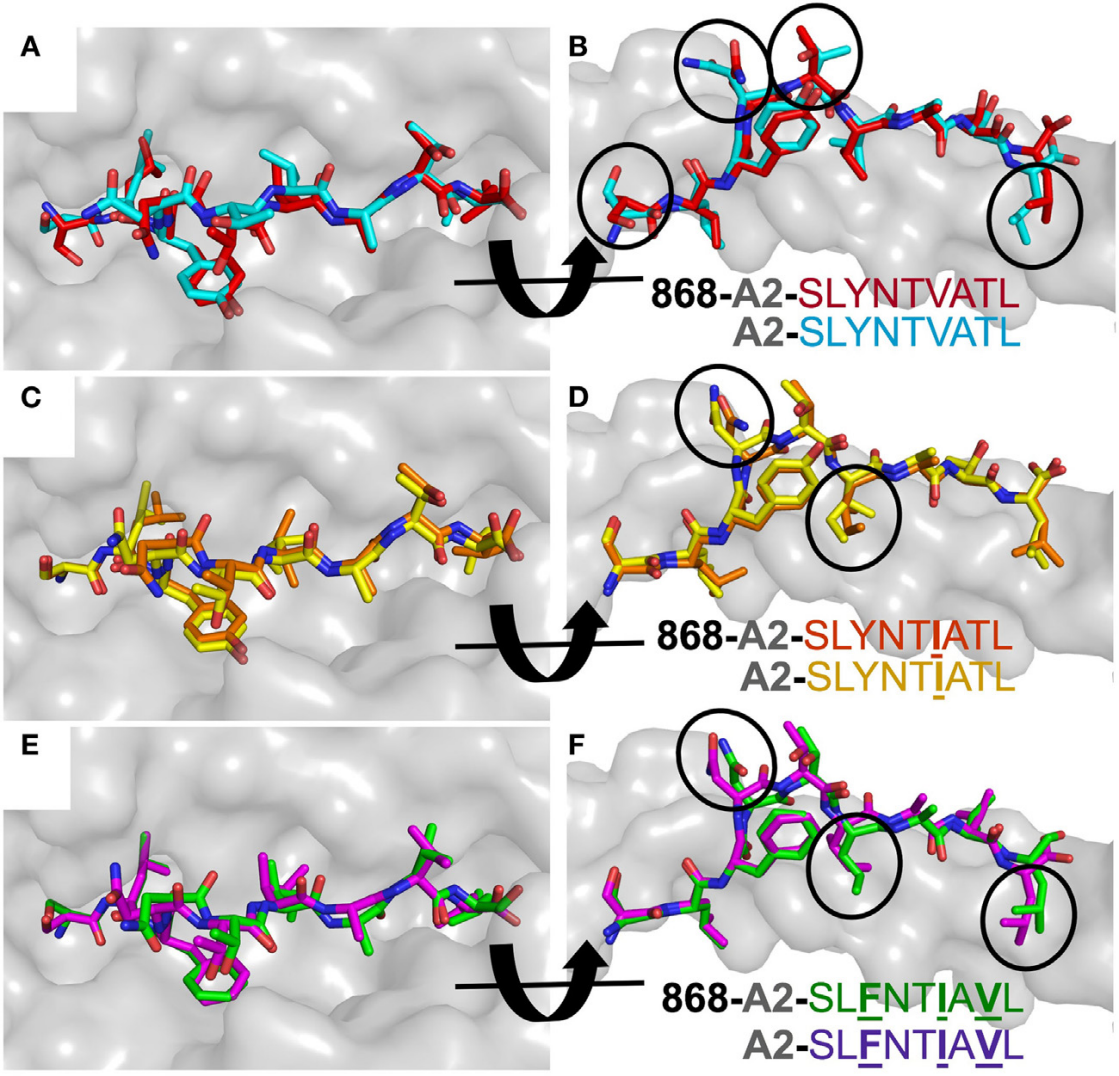
868 TCR binding alters the peptide conformation of the A2–SLYNTVATL escape mutants. Structural comparison of the A2–SLYNTVATL escape variants unligated and in complex with the 868 TCR. **(A,B)** A2–SLYNTVATL in complex with the 868 TCR (red sticks) vs. unligated A2–SLYNTVATL (cyan sticks). Small changes in the positions of the side chains that occur during TCR binding are circled. **(C,D)** A2–SLYNT**I**ATL (orange sticks) in complex with the 868 TCR vs. unligated A2–SLYNT**I**ATL (yellow sticks). Small changes in the positions of the side chains that occur during TCR binding are circled. **(E,F)** A2–SL**F**NT**I**A**V**L (green sticks) in complex with the 868 TCR vs. unligated A2–SL**F**NT**I**A**V**L (purple sticks). Small changes in the positions of the side chains that occur during TCR binding are circled.

Previous studies comparing SLYNTVATL and the SL**F**NTVATL mutated peptide showed that they adopted different conformations within the HLA A2 binding groove (7, 22). The T-cell recognition and on-rate kinetics with the G10 SL9-specific TCR in this study were very similar with A2–SLYNTVATL and A2–SL**F**NTVATL, leading the authors to conclude that one conformation, common to both peptides, was recognised by TCRs (22) and suggesting that TCR interactions with either A2–SLYNTVATL or A2–SL**F**NTVATL required an induced fit in the pMHC. Thus, SL9-derived peptides may bind to MHC dynamically and undergo conformational changes during TCR docking. Conformational peptide changes would likely elicit an entropic penalty during TCR ligation, not revealed by differences in contacts, and could contribute to weaker binding. However, based on previously solved structures [A2–SLYNTVATL (22), A2–SL**F**NTVATL (22), A2–SL**A**NTVATL (21), A2–SLYNT**I**ATL (21), A2–SLY**L**TVATL (21), A2–SLYN**V**VATL (21), A2–**A**LYNT**A**A**A**L (21), and A2–SL**F**NT**I**A**V**L (21)], some of which we replicated in this study, we show that A2–SLYNTVATL, A2–SLYNT**I**ATL, and A2–SL**F**NT**I**A**V**L all adopt a similar structure to every unbound SL9 peptide variant, apart from A2–SL**F**NTVATL (22), when ligated to the 868 TCR (Figure 4). These data make it possible to be more definitive about the direction of travel of the previously suggested induced fit model (22) (i.e., the odd one out is A2–SL**F**NTVATL and this peptide presumably adopts the more conventional structure for A2–SLYNTVATL type ligands prior to, or at the time of, TCR binding). Furthermore, these data do not support the notion that HIV escape is mediated by dynamic differences in ligand engagement during TCR binding to SL9 variants.

### The 868 TCR Adopts a Similar Energetic Strategy during Binding to SL9 Escape Variants

**Figure 5:**
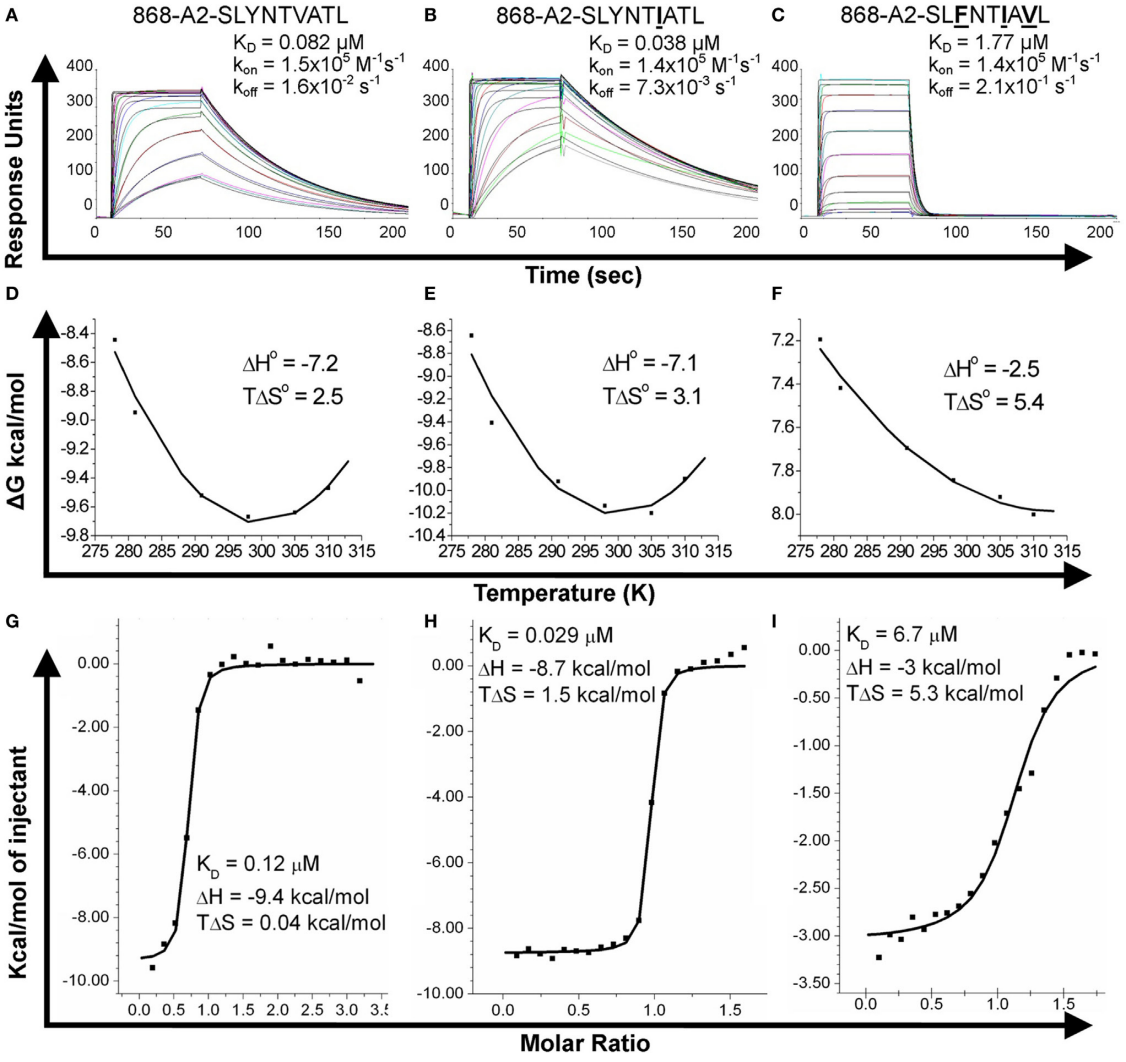

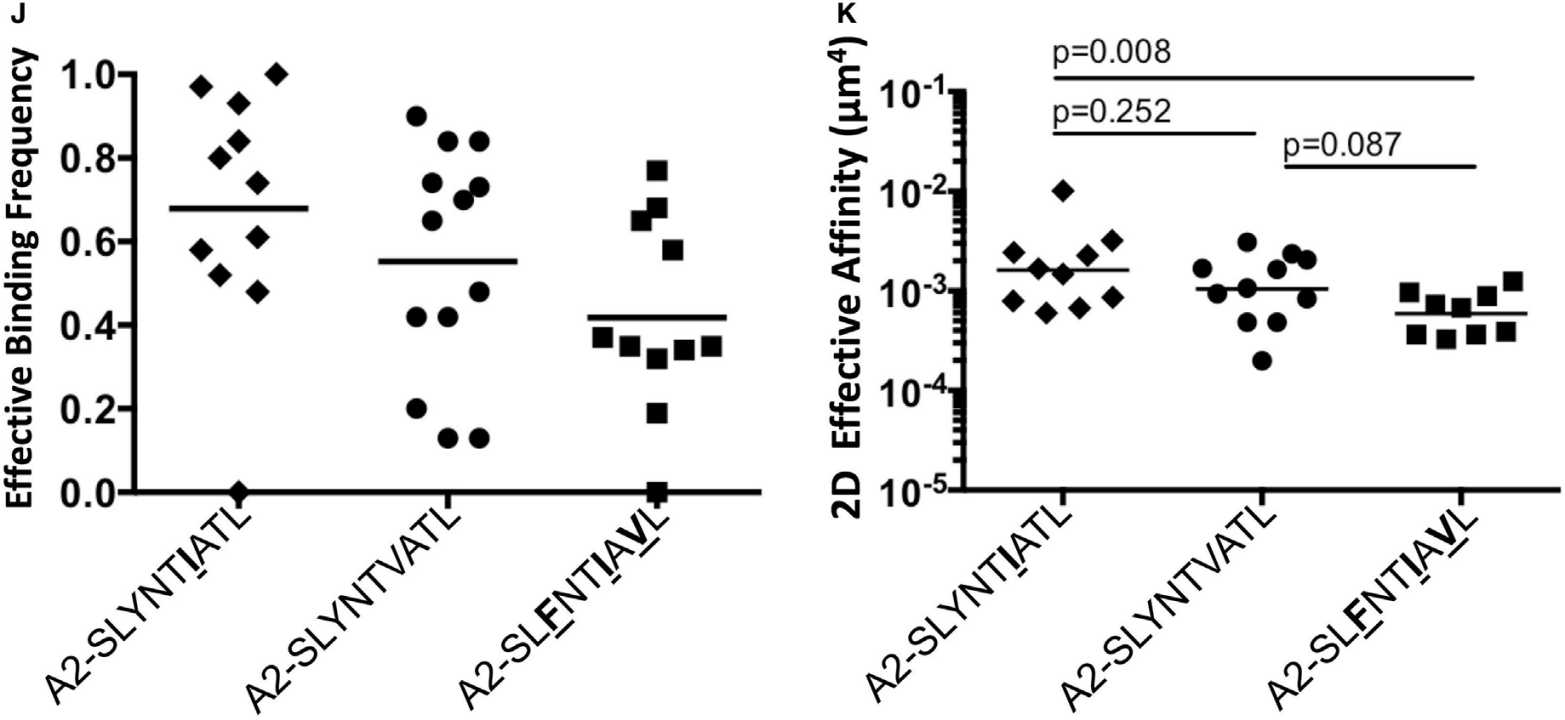
Binding affinity and thermodynamic analysis of the 868 TCR binding to A2–SLYNTVATL, A2–SLYNT**I**ATL, and **A2–**SL**F**NT**I**A**V**L. **(A–C)** Binding and kinetic analysis of the 868 TCR interaction with **(A)** A2–SLYNTVATL, **(B)** A2–SLYNT**I**ATL, and **(C)** A2–SL**F**NT**I**A**V**L. Experiments were performed independently using a BIAcore T100 equipped with a CM5 sensor chip and repeated in triplicate on different days using different protein preparations. Representative data are shown. **(D–F)** Thermodynamic analysis of 868 TCR with the aforementioned ligands and surface plasmon resonance (SPR) as above. Thermodynamic parameters were calculated according to the Gibbs–Helmholtz equation (Δ*G*° = Δ*H* − *T*Δ*S*°). The binding free energies, Δ*G*° (Δ*G*° = *RT*ln*K*_D_), were plotted against temperature (K) using non-linear regression to fit the three-parameter Van’t Hoff equation [*RT* ln *K*_D_ = Δ*H* − *T*Δ*S*° + ΔCp°(*T* − *T*_0_) − *T*ΔCp° ln (*T*/*T*_0_) with *T*_0_ = 298 K]. **(G–I)** Thermodynamic analysis of the 868 TCR–pMHC interaction was also performed using isothermal titration calorimetry 200 instrument to directly measure Δ*H*. These analyses show consistent values to those generated by SPR. Overall, the 868 TCR uses a generally similar binding mechanism and thermodynamic signature to interact with all three ligands. **(J)** Effective 2D binding frequency, using at least 5 cell pairs, and calculated as an average of 100 cell–cell contacts. **(K)** Effective 2D affinity (*A*_c_*K*_a_) calculated using adhesion frequency assays and reported in the text as geometric mean. Statistics were performed on log-transformed affinities and analysed with two-tailed, unpaired parametric *t*-tests and assumption of equal SDs.

**Figure 6:**
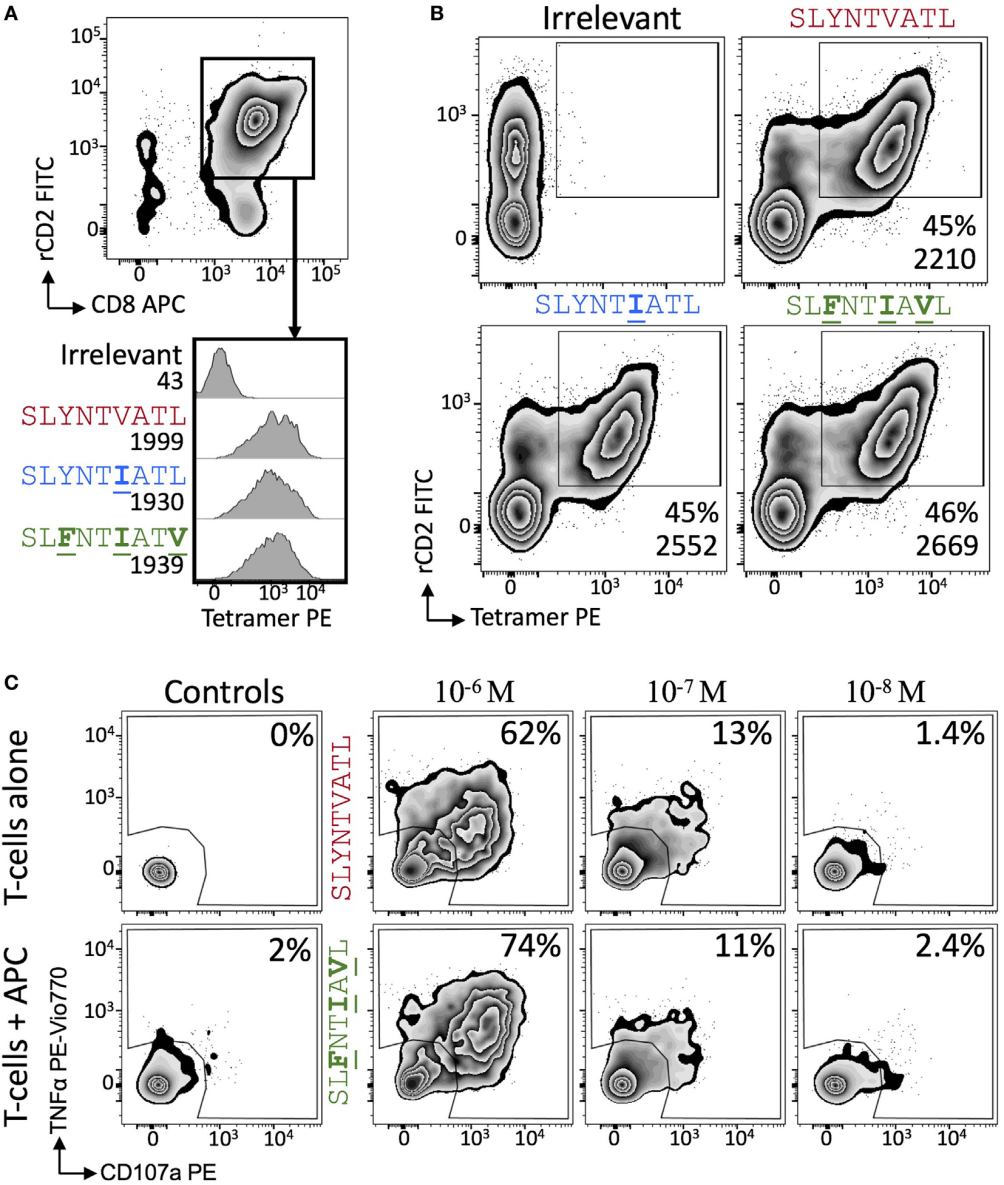
868 TCR-expressing T-cells bind escape mutant tetramers efficiently without the need for CD8. **(A)** Primary CD8^+^ T-cells were co-transduced with the 868 TCR and rat (r)CD2 prior to staining with 0.5 µg (with respect to MHC) of irrelevant (A2–ALWGPDPAAA), A2–SLYNTVATL, A2–SLYNT**I**ATL, and A2–SL**F**NT**I**A**V**L PE-conjugated peptide–MHC tetramers. Cells are gated on rCD2^+^CD8^+^ cells. Histograms show staining with indicated PE-conjugated tetramer with the mean fluorescence intensity (MFI) of this population displayed. **(B)** As for panel **(A)**, but 868 TCR and rat (r)CD2 were transduced into TCRβ chain negative Jurkat cells. MFI of tetramer staining is shown for cells in the rCD2^+^ tet^+^ gate. **(C)** 868 TCR transduced were incubated with T2 cells and either SLYNTVATL or SL**F**NT**I**A**V**L peptide at the concentrations shown for 5 h followed by the detection of CD107a and TNFα by flow cytometry.

We next explored the evidence for HIV escape being mediated by distinct TCR-binding parameters. It has been previously shown that the G10 SL9-specific TCR binds with a similar affinity to both A2–SLYNTVATL (*K*_D_ = 2.2 µM) and A2–SL**F**NTVATL (*K*_D_ = 5.2 µM) by SPR (22). Another study demonstrated that half maximal pMHC tetramer binding (DR_50_) to the SL9-specific D3 TCR was near identical for A2–SLYNTVATL, A2–SL**F**NTVATL, A2–SLYNTVA**V**L, A2–SL**F**NTVA**V**L, and A2–SL**F**NT**I**A**V**L (21). Finally, we have previously shown that the 868 TCR binds to a range of SL9 variants (11) (A2–SLYNTVATL, A2–SLYNT**I**ATL, A2–SLYNT**I**A**V**L, A2–SLYNTVA**V**L, A2–SL**F**NT**I**ATL, A2–SL**F**NTVA**V**L, A2–SL**F**NTVATL and A2–SL**F**NT**I**A**V**L). Consequently, the available data show that SL9-specific TCRs can engage SL9 variants within an affinity range that would be expected to easily activate T-cells. Here, we reproduced SPR-binding assays with the 868 TCR for the three ligands included in this study. These data demonstrated that the 868 TCR bound to A2–SLYNTVATL with a *K*_D_ of 82 nM (Figure 5A) confirming that this is, by far, the strongest binding natural TCR–peptide–MHC interaction ever recorded (51, 52). Indeed, the affinity was even greater with the A2–SLYNT**I**ATL variant (*K*_D_ = 38 nM; Figure 5B) that dominated in the patient at the time the blood sample was taken that was used to generate the 868 T-cell line (9, 10). 868 TCR interactions with A2–SLYNTVATL and the A2–SLYNT**I**ATL variant were characterised by a relatively slow off-rate (*k*_off_ = 1.6 × 10^−2^–7.3 × 10^−3^ s^−1^) compared to the majority of other natural TCR–pMHC interactions (average *k*_off_ ~ 2 × 10^−1^ s^−1^) (51). Although the strength of the 868-A2–SL**F**NT**I**A**V**L interaction was 21-fold and 45-fold weaker than for A2–SLYNTVATL or A2–SLYNT**I**ATL, respectively, due to a much faster off-rate (*k*_off_ = 2.1 × 10^−1^ s^−1^), the affinity (*K*_D_ of 1.77 µM; Figure 5C) was still in the range of the very best natural anti-pathogen TCRs (52, 53). Thus, this weaker affinity alone was unlikely to explain the ability of the SL**F**NT**I**A**V**L variant to evade T-cell detection. Indeed, we have described a functional HLA A2-restricted TCR that binds its cognate pMHC with over 100-fold weaker affinity than the 868 TCR and A2–SL**F**NT**I**A**V**L (54, 55). To get a more complete picture of how the 868 TCR engages its cognate ligands we also undertook thermodynamic analyses by SPR (Figures 5D–F; Table 4) and directly by isothermal calorimetry (ITC; Figures 5G–I). The results of these independent techniques correlated well and showed that 868 TCR engagement was driven by energetically favourable enthalpy and entropy changes to all three ligands. Thus, for the 868 TCR, there was a net increase in the number of electrostatic interactions and a transition from order-disorder during binding. Considering that the 868 TCR CDR3 loops underwent similarly large conformational changes during engagement to all three SL9 variants studied here (shown for 868-A2–SLY interaction in Figure 1C), a movement that would probably be entropically unfavourable, the expulsion of ordered water molecules from the interface, and/or order–disorder transitions in other parts of the molecules presumably contributed to the net favourable entropic change of the interaction. The lower affinity of 868 TCR for A2–SL**F**NT**I**A**V**L compared to A2–SLYNTVATL or A2–SLYNT**I**ATL was associated with a much reduced enthalpic component, but more favourable entropy. We used 2D binding analysis to confirm our observations using SPR (Figures 5J,K). In accordance with our previous findings with other TCRs (55, 56), we observed an identical relationship between the strength of interaction between 868 and the three SL9-derived ligands by 2D analysis and SPR. 868-A2–SLYNT**I**ATL was characterised by the strongest 2D effective affinity (1.6 × 10^−3^ μm^4^), followed by 868-A2–SLYNTVATL (1.0 × 10^−3^ μm^4^), with 868-A2–SL**F**NT**I**A**V**L generating the weakest effective 2D affinity (5.9 × 10^−4^ μm^4^). To further confirm our observations from 2D and 3D binding experiments, we next examined the binding of pMHC tetramers to the 868 TCR at the T-cell surface. Staining of 868 TCR-transduced CD8^+^ primary T-cells was similar for tetramers made with A2–SLYNTVATL, A2–SLYNT**I**ATL, and A2–SL**F**NT**I**A**V**L (Figure 6A). The CD8 coreceptor is known to bind to a site on MHC class I distinct from the TCR docking platform and cooperates with the TCR to aid the engagement of pMHC tetramers (57) and the absence of CD8 binding is known to raise the TCR affinity threshold required for tetramer staining (58, 59). 868 TCR^+^ Jurkat T-cells, that do not express CD8, also stained equally well with A2–SLYNTVATL, A2–SLYNT**I**ATL, and A2–SL**F**NT**I**A**V**L tetramers (Figure 6B). Given our observations that the TCR affinity threshold required for T-cell activation is considerably lower than that required for pMHC tetramer binding (58) and finding that these reagents can even fail to stain fully functional T-cells (47, 60), our results suggest that the SL**F**NT**I**A**V**L ultimate escape variant would still be a very efficient T-cell agonist. Indeed, the 868 TCR-transduced primary CD8^+^ T-cells exhibited similar reactivity to SLYNTVATL and SL**F**NT**I**A**V**L peptides when using standard T-cell activation assay whereby T-cells, antigen-presenting cells, and peptide are added directly to tissue culture wells and allowed to incubate (Figure 6C). We conclude that while there is a substantial reduction of TCR binding to the SL**F**NT**I**A**V**L triple mutant sequence, this reduction cannot explain the observed immune escape *per se* as assumed by all previous studies in this system (6, 7, 11, 23).

**Table 4:** Affinity and kinetic measurements of 868-A2–SLYNTVATL, 868-A2–SLYNTIATL, and 868-A2–SLFNTIAVL at different temperatures.

MHC–peptide	Temp (°C)	*k*_on_ (M^**−**1^S^**−**1^)	*k*_off_ (S^**−**1^)	$k_{\text{D}}^{\text{kinj}}$ (μM)	$k_{\text{D}}^{\text{Equl}}$ (μM)
A2–SLYNTVATL	5	1.1 × 10^4^	2.2 × 10^−3^	0.2	0.23
	8	2.8 × 10^4^	3.7 × 10^−3^	0.13	0.11
	18	7.1 × 10^4^	7.1 × 10^−3^	0.1	0.072
	25	1.5 × 10^5^	1.6 × 10^−2^	0.11	0.082
	32	4.3 × 10^5^	5.6 × 10^−2^	0.13	0.124
	37	4.0 × 10^5^	6.9 × 10^−2^	0.17	0.21

A2–SLYNT**I**ATL	5	9.2 × 10^3^	1.3 × 10^−3^	0.13	0.16
	8	5.4 × 10^4^	2.7 × 10^−3^	0.05	0.048
	18	7.6 × 10^4^	4.2 × 10^−3^	0.055	0.035
	25	1.4 × 10^5^	7.3 × 10^−3^	0.052	0.038
	32	3.1 × 10^5^	1.9 × 10^−2^	0.06	0.049
	37	6.4 × 10^5^	3.2 × 10^−2^	0.05	0.11

A2–SL**F**NT**I**A**V**L	5	2.3 × 10^4^	7.7 × 10^−2^	3.3	2.2
	8	6.3 × 10^4^	1.1 × 10^−1^	1.7	1.7
	18	1.3 × 10^5^	1.8 × 10^−1^	1.42	1.65
	25	1.4 × 10^5^	2.1 × 10^−1^	1.48	1.77
	32	1.6 × 10^5^	3.0 × 10^−1^	1.9	2.11
	37	1.0 × 10^6^	5.3 × 10^−1^	0.53	2.3

The other way by which peptides can escape from host CTL surveillance is by mutating MHC anchors to reduce binding and presentation at the infected cell surface. However, previous studies have shown that the SL**F**NT**I**A**V**L peptide binds to HLA A2 with 50–400% of the affinity the index SL9 sequence depending on the study (Table 5). Such minor relative differences were not thought to be able to explain how the triple mutant peptide escapes from CTL (6, 7, 9, 18). Our own studies with a modified version of the 868 TCR that recognises the escape variants well showed that this TCR could recognise CD4^+^ T-cells infected with primary HIV isolates carrying known escape variants (11). Thus, these variants must be presented at the cell surface in the context of HLA A2. Importantly, these variants were not recognised by T-cells expressing the wild-type 868 TCR, thus verifying that these viruses are indeed escape mutants (11). Consequently, the established literature indicates that the common variant peptides in this system bind sufficiently well to HLA A2 to be presented (6, 7, 9, 18) and sufficiently well to the 868 and D3 TCRs to be recognised by T-cells expressing these SL9-specific receptors. These apparently incongruous findings prompted us to next re-examine peptide binding in the SL9 system.

**Table 5:** Relative binding of SLYNTVATL and SLFNTIAVL to HLA A*0201.

SLYNTVATL	SLFNTIAVL	Method
100%	48%	S35 methionine pulse chase (9)
100% (*K*_D_ = 50 nM)	53% (*K*_D_ = 94 nM)	Direct competition with radio-labelled ligand (18)
100% (*K*_D_ = 300 nM)	300% (*K*_D_ = 100 nM)	Quantitative HLA A2 ELISA (6)
100% (*K*_D_ ~ 3.5 nM)	400% (*K*_D_ ~ 0.8 nM)	Quantitative HLA A2 ELISA (7)

### CTL Escape by the Ultimate SL9 Escape Variant Mediated by a Fast Peptide Off-Rate

**Figure 7:**
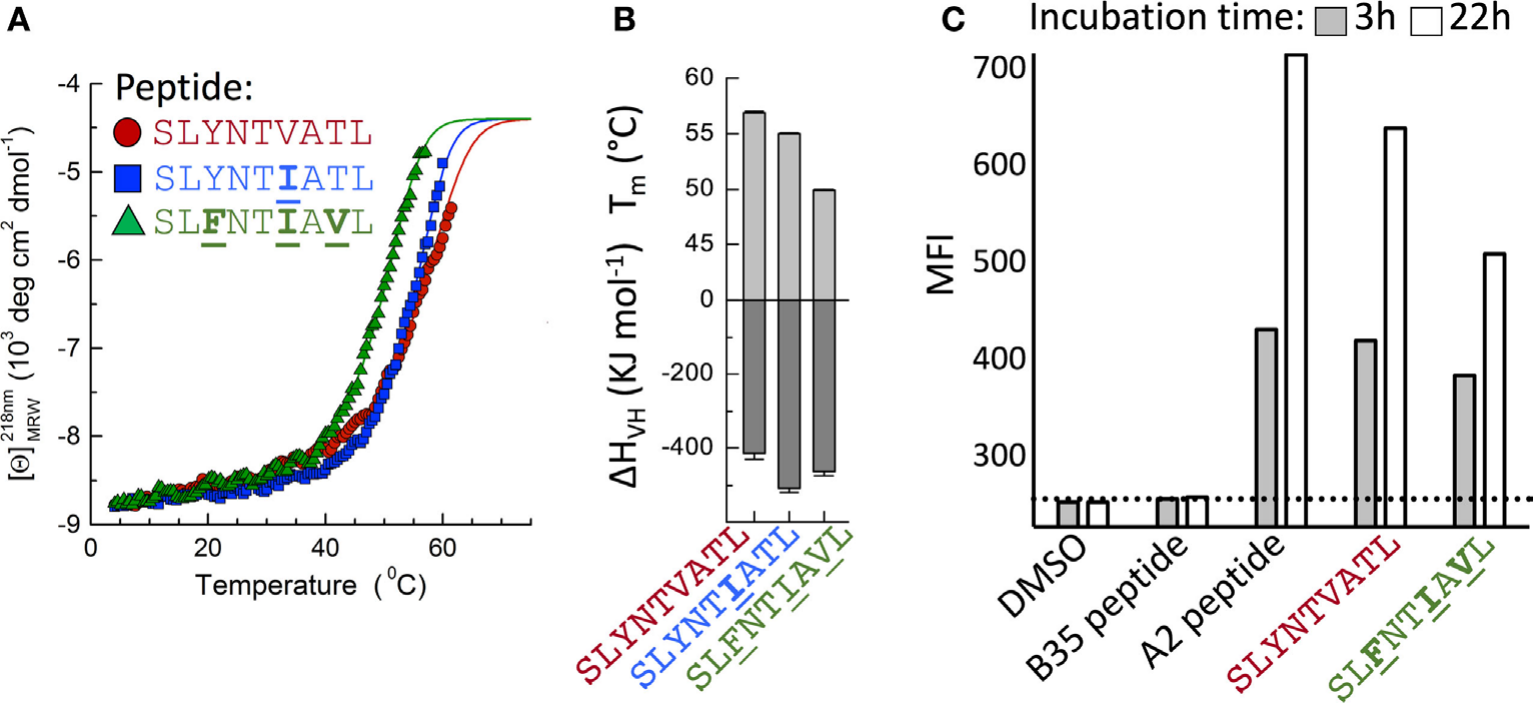
Stability of A2–SLYNTVATL, **A2–**SLYNT**I**ATL, and **A2–**SL**F**NT**I**A**V**L. **(A)** CD thermal denaturation curves recorded at 218 nm are shown for selected samples as indicated. Dots represent measured values fitted assuming a two-state trimer-to-monomer transition (solid lines) as described in Section “Materials and Methods.” **(B)** Bar graphs of the thermal stability with respect to melting temperature (upper) and van’t Hoff’s enthalpy of unfolding (lower panel). Error bars in panels **(A,B)** represent SD resulting from the multivariable curve fitting of data from one experiment with each peptide. **(C)** T2 cells incubated for 3 or 22 h with 10^−5^ M of each peptide [Influenza (Flu) Matrix, GILGFVFTL, HIV Gag wild-type SLYNTVATL, and Gag triple mutant SL**F**NT**I**A**V**L] or DMSO control were stained for HLA-A2 and fixed. The dashed line is set at the mean fluorescence intensity (MFI) of the negative control peptide (EBV HPVGEADYFEY that binds HLA B*3501).

**Figure 8:**
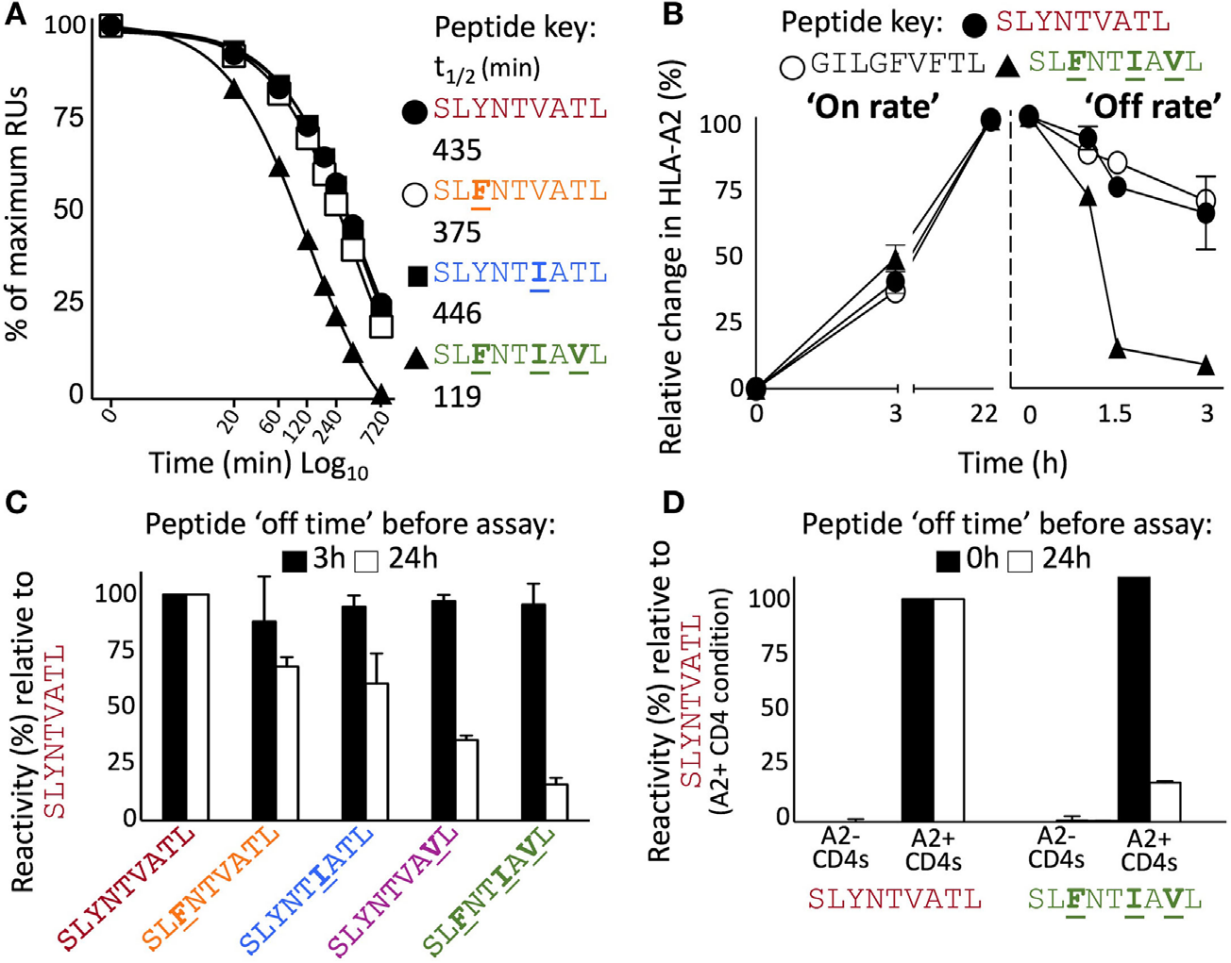
Instability at the cell surface mediates viral escape by the triple mutant epitope, A2**–**SL**F**NT**I**A**V**L. **(A)** Relative binding of 868 TCR to A2–peptide complexes by SPR over time. The SPR chip was maintained at 37°C. Functional half-life was determined by injecting the 868 TCR at a fixed concentration of 100 µM over 720 min and recording the drop in total RUs over 8 different time points. A faster drop in RUs (converted into a *T*_1/2_) was interpreted to indicate greater peptide instability, detected by loss of binding by the antigen-specific 868 TCR. **(B)** Left panel shows relative HLA-A2 expression following incubation (0, 3, and 22 h) of T2 cells with indicated peptide. Results are normalised to the maximum mean fluorescence intensity seen for each peptide. The right panel shows T2 cells incubated with peptide for 22 h prior to being washed and cultured in an excess of AIM-V media for the indicated times prior to staining and fixing. Data displayed as HLA-A2 expression relative to maximum seen at 22 h. **(C)** T2 cells were pulsed for 1 h with the peptides shown, washed extensively, and then cultured in an excess of media for 3 h (black bar) or 24 h (open bar) before being co-incubated for 5 h with T-cells from two donors transduced with the 868 TCR. CD107a and TNFα were used to establish percentage reactivity by flow cytometry. Values were normalised relative to SLYNTVATL peptide. SEM is shown for two different donor T-cells. Untransduced CD8^+^ T-cells did not respond to peptide (data not shown). Raw data for panel **(C)** is shown in Figure S1 in Supplementary Material. **(D)** As panel **(C)** but using primary HLA-A2^−^ and HLA-A2^+^ CD4^+^ T-cells as targets and culturing in excess media for either 0 or 24 h prior to assay. Raw data for panel **(D)** is shown in Figure S2 in Supplementary Material.

Detailed analysis of the thermal melting of A2–SLYNTVATL, A2–SLYNT**I**ATL, and A2–SL**F**NT**I**A**V**L by circular dichroism showed the melting temperature (*T*_m_) of the wild-type peptide was higher than that of the triple mutant (57°C compared to 49°C; Figures 7A,B) suggesting that the triple mutant is less stable. These data are in accordance with two previous studies showing that SL**F**NT**I**A**V**L binds to HLA A2 with ~50% reduced affinity compared to SL9 (9, 18) but at odds with two studies indicating that SL**F**NT**I**A**V**L is the better ligand (6, 7). Peptide binding to HLA A2 has not previously been thought to result in the immune escape observed with this epitope, which instead has been attributed to a lack of recognition by SL9-specific TCRs (6, 7, 11, 23). Stabilisation of HLA A2 at the T2 cell surface in the continuous presence of 100 µM peptide (steady-state binding; Figure 7C) showed that the SL**F**NT**I**A**V**L peptide stabilised HLA A2 with 89% of that seen with the wild-type SL9 peptide after 3 h. This relatively minor difference increased in an overnight assay where stabilisation with SL**F**NT**I**A**V**L was ~65% of that seen with the wild-type peptide. Our previous examinations of HLA A2 peptide stability by SPR have shown that good agonists produce pMHC with a half-life of >6 h in a cell free system (61). Similar experiments, performed by examining the relative binding of 868 TCR to A2–peptide by SPR over a time course at 37°C, showed that both A2–SLYNTVATL and A2–SLYNT**I**ATL had a half-life of >7 h. By contrast, the half-life of A2–SL**F**NT**I**A**V**L on the same chip was <2 h (Figure 8A). We thus reasoned that the stabilisation of peptide–HLA measured in a short-term binding assay like those previously used in this system (9, 18) would be determined more by peptide *on-rate* than the peptide *off-rate* or dwell time. To further test this possibility, we examined the “on rate” of peptide in T2 cell stabilisation assays. Relative stabilisation was similar for the SLYNTVATL, SL**F**NT**I**A**V**L and the GILGFVFTL influenza matrix-derived peptide that is often used as a positive control for HLA A2 binding (Figure 8B: left panel). We also examined the stability of these A2–peptide complexes at the cell surface by monitoring the amount of HLA A2 on T2 cells incubated with each peptide prior to washing and further culture. The vast majority of HLA A2 stabilised with GILGFVFTL or SLYNTVATL peptide was still present at the cell surface 3 h after the peptide was removed from the assay by cell washing. In stark contrast, most of the HLA A2 stabilised by the SL**F**NT**I**A**V**L peptide had decayed in half this time (Figure 8B: right panel). Collectively, these binding data explain how previous studies, using short-term binding assays in the continuous presence of peptide, have shown minor differences in the binding of SL9 and SL**F**NT**I**A**V**L escape variant (Table 5). They also suggest that the dominant mechanism by which the SL**F**NT**I**A**V**L mutant allows immune escape is due to this peptide producing a much shorter-lived peptide–HLA complex. This reduced half-life of antigen at the infected cell surface would be expected to considerably reduce the antigen density on infected cells and thereby facilitate immune escape. To test this notion, we next examined how long peptide pulsed cells could remain as T-cell targets for CD8^+^ T-cells transduced with the wild-type 868 TCR. These revealing experiments allowed examination of the combined effects of differences in TCR binding and HLA stability in a relevant biological context. Cells pulsed with the SLYNTVATL remained effective targets for 868 TCR-expressing CD8^+^ T-cells after 24 h of culture (Figure 8C; Figure S1 in Supplementary Material). By contrast, targets pulsed with the SL**F**NTVATL, SLYNT**I**ATL, or SLYNTVA**V**L variant peptides showed reduced levels of recognition after 24 h. The SL**F**NT**I**A**V**L triple mutant sequence produced the largest effect and was recognised well if experiments were performed immediately but recognition dropped to less than 20% of that seen for the SLYNTVATL peptide at *t*_0_, after targets were cultured for 24 h prior to the T-cell recognition assay. Similar data were observed when primary CD4^+^ T-cells, which form the major reservoir for HIV virus *in vivo*, were used as targets (Figure 8D). Relative to SLYNTVATL, the SL**F**NT**I**A**V**L pulsed cells made better targets at *t*_0_ (125%) but their ability to activate the 868 TCR cells diminished to 18% when allowed to culture for 24 h prior to assay (Figure 8D; Figure S2 in Supplementary Material). These results suggest that the major mechanism by which SL**F**NT**I**A**V**L escapes from CTL is through loss of peptide–HLA stability, although reduced TCR interaction with this mutant is also likely to play a secondary role.

## Discussion

Recognition and escape from the HLA A2-restricted HIV epitope SL9 has been examined in well over 50 studies to date, but major questions are still outstanding in this system as there has been no TCR–A2–SL9 co-complex structure. Here, we solved the structure of the free 868 TCR, a TCR first described almost 20 years ago (10), and the structure of this TCR in complex with A2–SLYNTVATL. Comparison of the free and ligated TCR structures suggested an “induced fit” mechanism that involved major structural reorganisation of the antigen binding face of the TCR. The CDR3 loops made the biggest movement (almost 7 Å for CDR3α and >3 Å for CDR3β) to avoid steric clashes with the central portion of the peptide, while the CDR1 and CDR2 loops interacted mainly with MHC *via* a more “lock-and-key” interaction.

It has been known for over a decade that that the SLYNTVATL and SL**F**NTVATL bind to HLA A2 with very different conformations, but engage the G10 TCR with near identical on-rates, leading the authors to conclude that one conformation, common to both peptides, was recognised by TCRs (22). Since A2–SLYNTVATL does not change upon TCR binding, we can now be confident that the conformation adopted by this, and many other SL9 variants (21), dominates during TCR recognition. Thus, the evidence suggests that A2–SL**F**NTVATL undergoes an induced fit upon TCR binding (22) although conclusive proof will await a TCR-A2–SL**F**NTVATL structure. We further solved the co-complex structure of the 868 TCR with the A2–SLYNT**I**ATL (the variant that predominated in patient 868 in 1996 when the patient blood sample from which this T-cell clone was grown was taken), and the structure of the 868 TCR in complex with the A2–SL**F**NT**I**A**V**L, the ultimate escape variant in this system. Resolution of the latter structure came as a surprise as residues that mutate in positions 3, 6, and 8 to produce this mutant are known to be solvent exposed and had been assumed to act as prominent TCR contacts (6, 7, 21–23). In contrast to this prevailing hypothesis we found that the asparagine at position 4 was the dominant contact residue. Indeed, there were more contacts with this residue than with the position 3 tyrosine, position 6 valine, and position 8 threonine combined. An alanine mutation scan across the peptide backbone showed that while positions 4 and 5 in SLYNTVATL were important for recognition, the most severe non-MHC anchor change was Y3A. In accordance, the position 3Y sub-library also gave the strongest response of the 180 sub-libraries during a 9-mer positional scanning combinatorial library screen of T-cells transduced with the 868 TCR (62). The importance of the tyrosine residue at position 3 is consistent with our previous observation that large bulky amino acids at this position in HLA A2-bound peptides can form a bridge with more C-terminal residues (usually position 5) (63). Replacing residue 3 with a smaller alanine side chain can abolish this important intra-peptide stabilisation and have knock-on effects at peptide residue 4 that abrogate TCR binding. Thus, our findings here point towards a common mechanism of peptide presentation by HLA A2 in which peptide residue 3 can be essential for maintaining antigenic identity.

We next undertook a detailed biophysical and thermodynamic study of 868 TCR binding to A2–SLYNTVATL, A2–SLYNT**I**ATL, and A2–SL**F**NT**I**A**V**L. The 868 TCR exhibited, by far, the strongest affinity of a peptide–HLA ligand for a natural TCR (52) and bound to A2–SLYNTVATL and A2–SLYNT**I**ATL ligands with *K*_Ds_ of 82 and 38 nM, respectively, or over 10× stronger binding than the next highest TCR–pMHC interaction measured by BIAcore (52). The median affinity for human antiviral TCR–pMHC interactions is *K*_D_ ~5 μM (52). 868 TCR binding was characterised by a relatively slow off-rate from the A2–SLYNTVATL and A2–SLYNT**I**ATL ligands. The other SL9-TCR that has been examined by SPR, G10, had an off-rate 3.75 times faster than 868, and a 4.5 times slower on rate, resulting in a weaker affinity of *K*_D_ of 2.2 µM for A2–SLYNTVATL compared to 868 (22). While binding of the 868 TCR was much reduced for A2–SL**F**NT**I**A**V**L compared to the other ligands, it still had a very respectable affinity (*K*_D_ = 1.77 μM) that falls at the higher end of the spectrum for TCR agonists (52).

The mode of 868 TCR engagement and its affinity with the A2–SL**F**NT**I**A**V**L ultimate escape variant in this system did not lend full support to the prevailing belief that loss of recognition of SL9 occurs mainly *via* interfering with TCR binding. The fact that CD8^+^ T-cells transduced with the 868 TCR could recognise SL**F**NT**I**A**V**L pulsed target cells well and stained with A2–SL**F**NT**I**A**V**L tetramers was also a surprise, but in accordance with an earlier study that showed that A2–SL**F**NT**I**A**V**L bound to the D3 TCR as well as A2–SLYNTVATL in tetrameric form and that the SL**F**NT**I**A**V**L concentration required for half maximal lysis was a highly respectable 4 × 10^−10^ M (21). In further accordance with positions 3, 6, and 8 not dominating A2–SL9-specific TCR binding, the position 4 asparagine sub-library was by far the most potent of the 180 sub-mixtures for activating T-cell clone 003 known to express a different SL9-specific TCR (62). These combined data do not support the notion that the SL**F**NT**I**A**V**L escapes from CTL *via* lack of TCR binding, prompting us to take a closer look at the MHC binding of ligands in this system. Data from three different studies using three different methodologies show that SL**F**NT**I**A**V**L binds to HLA A2 with 50–400% of the affinity of SLYNTVATL. Our previous SPR experiments have shown that good agonists produce pMHC with a half-life of >6 h in a cell-free system (61). Indeed, our data here show that both A2–SLYNTVATL and A2–SLYNT**I**ATL had half-lives of over 7 h on an SPR chip maintained at 37°C compared to <2 h for A2–SL**F**NT**I**A**V**L. We reasoned that the peptide-binding assays that have been performed previously in this system, including by ourselves (9), would be most influenced by peptide *on-rate* but that the biology would be most linked to peptide *off-rate* at the target cell surface. HLA A2^+^ target cells pulsed with SLYNTVATL peptide remained effective targets for SL9-specific CD8^+^ T-cells after 24 h of culture as has been observed with a different HIV Gag-derived epitope (27). The SL**F**NTVATL, SLYNT**I**ATL and SLYNTVA**V**L peptides [which bind to the 868 TCR with dissociation constants of 2.9 µM, <100 nM, and ~300 nM, respectively (52)] were seen well when targets presenting these ligands were used immediately for T-cell assays but exhibited only 67, 60, and 35% recognition of that observed with SLYNTVATL peptide when targets were cultured for 24 h prior to the T-cell assay. The biggest effect was observed with the SL**F**NT**I**A**V**L where >80% of the activity was lost after 24 h of culture. These results were supported by other experiments looking the stability of the A2–SL**F**NT**I**A**V**L complex in comparison to other ligands.

Collectively, our results suggest that the instability of the A2–SL**F**NT**I**A**V**L molecule is likely to provide the major mechanism of escape from SL9-specific CTL. The accumulation of mutations at positions 3, 6, and 8 to fixation over a 10-year period (6, 7) demonstrates the extraordinary lengths that HIV will go to in order to avoid detection by these key antiviral cells. Our study further highlights that the results of short-term, steady-state peptide-binding assays can be misleading and suggests that future studies should employ more relevant biological readouts.

## Author Contributions

DC, AF, GD, EZ, ML, KM, JLB, FM, CH, AB, JSB, JM, AJAS, KB, BE, and PR performed and/or directed experiments, analysed data, and critiqued the manuscript. DC and AKS conceived, funded, and directed the project and wrote the manuscript.

## Conflict of Interest Statement

The authors declare that the research was conducted in the absence of any commercial or financial relationships that could be construed as a potential conflict of interest.

## References

[1] Sewell AK Price DA Oxenius A Kelleher AD Phillips RE Cytotoxic T lymphocyte responses to human immunodeficiency virus: control and escape. Stem Cells (2000) 18:230–44. 10.1634/stemcells.18-4-230 10924089

[2] Goulder PJ Watkins DI HIV and SIV CTL escape: implications for vaccine design. Nat Rev Immunol (2004) 4:630–40. 10.1038/nri1417 15286729

[3] Carlson JM Le AQ Shahid A Brumme ZL. HIV-1 adaptation to HLA: a window into virus-host immune interactions. Trends Microbiol (2015) 23:212–24. 10.1016/j.tim.2014.12.008 25613992

[4] Kaslow RA Carrington M Apple R Park L Munoz A Saah AJ Influence of combinations of human major histocompatibility complex genes on the course of HIV-1 infection. Nat Med (1996) 2:405–11. 10.1038/nm0496-405 8597949

[5] Browning M Krausa P Genetic diversity of HLA-A2: evolutionary and functional significance. Immunol Today (1996) 17:165–70. 10.1016/0167-5699(96)80614-1 8871347

[6] Iversen AK Stewart-Jones G Learn GH Christie N Sylvester-Hviid C Armitage AE Conflicting selective forces affect T cell receptor contacts in an immunodominant human immunodeficiency virus epitope. Nat Immunol (2006) 7:179–89. 10.1038/ni1298 16388312

[7] Tenzer S Wee E Burgevin A Stewart-Jones G Friis L Lamberth K Antigen processing influences HIV-specific cytotoxic T lymphocyte immunodominance. Nat Immunol (2009) 10:636–46. 10.1038/ni.1728 19412183

[8] Purbhoo MA Sewell AK Klenerman P Goulder PJ Hilyard KL Bell JI Copresentation of natural HIV-1 agonist and antagonist ligands fails to induce the T cell receptor signaling cascade. Proc Natl Acad Sci U S A (1998) 95:4527–32. 10.1073/pnas.95.8.4527 9539771 PMC22523

[9] Sewell AK Harcourt GC Goulder PJ Price DA Phillips RE Antagonism of cytotoxic T lymphocyte-mediated lysis by natural HIV-1 altered peptide ligands requires simultaneous presentation of agonist and antagonist peptides. Eur J Immunol (1997) 27:2323–9. 10.1002/eji.1830270929 9341776

[10] Wilson JD Ogg GS Allen RL Goulder PJ Kelleher A Sewell AK Oligoclonal expansions of CD8(+) T cells in chronic HIV infection are antigen specific. J Exp Med (1998) 188:785–90. 10.1084/jem.188.4.785 9705961 PMC2213349

[11] Varela-Rohena A Molloy PE Dunn SM Li Y Suhoski MM Carroll RG Control of HIV-1 immune escape by CD8 T cells expressing enhanced T-cell receptor. Nat Med (2008) 14:1390–5. 10.1038/nm.1779 18997777 PMC3008216

[12] Tsomides TJ Aldovini A Johnson RP Walker BD Young RA Eisen HN. Naturally processed viral peptides recognized by cytotoxic T lymphocytes on cells chronically infected by human immunodeficiency virus type 1. J Exp Med (1994) 180:1283–93. 10.1084/jem.180.4.1283 7523570 PMC2191672

[13] Goulder PJ Altfeld MA Rosenberg ES Nguyen T Tang Y Eldridge RL Substantial differences in specificity of HIV-specific cytotoxic T cells in acute and chronic HIV infection. J Exp Med (2001) 193:181–94. 10.1084/jem.193.2.181 11148222 PMC2193346

[14] Ogg GS Jin X Bonhoeffer S Dunbar PR Nowak MA Monard S Quantitation of HIV-1-specific cytotoxic T lymphocytes and plasma load of viral RNA. Science (1998) 279:2103–6. 10.1126/science.279.5359.2103 9516110

[15] Ogg GS Kostense S Klein MR Jurriaans S Hamann D McMichael AJ Longitudinal phenotypic analysis of human immunodeficiency virus type 1-specific cytotoxic T lymphocytes: correlation with disease progression. J Virol (1999) 73:9153–60. 10516022 10.1128/jvi.73.11.9153-9160.1999 PMC112948

[16] Christie NM Willer DO Lobritz MA Chan JK Arts EJ Ostrowski MA Viral fitness implications of variation within an immunodominant CD8+ T-cell epitope of HIV-1. Virology (2009) 388:137–46. 10.1016/j.virol.2009.03.003 19368950

[17] Edwards CT Pfafferott KJ Goulder PJ Phillips RE Holmes EC. Intrapatient escape in the A*0201-restricted epitope SLYNTVATL drives evolution of human immunodeficiency virus type 1 at the population level. J Virol (2005) 79:9363–6. 10.1128/JVI.79.14.9363-9366.2005 15994836 PMC1168780

[18] Brander C Hartman KE Trocha AK Jones NG Johnson RP Korber B Lack of strong immune selection pressure by the immunodominant, HLA-A*0201-restricted cytotoxic T lymphocyte response in chronic human immunodeficiency virus-1 infection. J Clin Invest (1998) 101:2559–66. 10.1172/JCI2405 9616227 PMC508845

[19] Goulder PJ Sewell AK Lalloo DG Price DA Whelan JA Evans J Patterns of immunodominance in HIV-1-specific cytotoxic T lymphocyte responses in two human histocompatibility leukocyte antigens (HLA)-identical siblings with HLA-A*0201 are influenced by epitope mutation. J Exp Med (1997) 185:1423–33. 10.1084/jem.185.8.1423 9126923 PMC2196285

[20] Douek DC Betts MR Brenchley JM Hill BJ Ambrozak DR Ngai KL A novel approach to the analysis of specificity, clonality, and frequency of HIV-specific T cell responses reveals a potential mechanism for control of viral escape. J Immunol (2002) 168:3099–104. 10.4049/jimmunol.168.6.3099 11884484

[21] Martinez-Hackert E Anikeeva N Kalams SA Walker BD Hendrickson WA Sykulev Y. Structural basis for degenerate recognition of natural HIV peptide variants by cytotoxic lymphocytes. J Biol Chem (2006) 281:20205–12. 10.1074/jbc.M601934200 16702212

[22] Lee JK Stewart-Jones G Dong T Harlos K Di Gleria K Dorrell L T cell cross-reactivity and conformational changes during TCR engagement. J Exp Med (2004) 200:1455–66. 10.1084/jem.20041251 15583017 PMC2211951

[23] Jamieson BD Yang OO Hultin L Hausner MA Hultin P Matud J Epitope escape mutation and decay of human immunodeficiency virus type 1-specific CTL responses. J Immunol (2003) 171:5372–9. 10.4049/jimmunol.171.10.5372 14607940

[24] Morikawa Y Zhang WH Hockley DJ Nermut MV Jones IM. Detection of a trimeric human immunodeficiency virus type 1 Gag intermediate is dependent on sequences in the matrix protein, p17. J Virol (1998) 72:7659–63. 9696871 10.1128/jvi.72.9.7659-7663.1998 PMC110034

[25] Kelleher AD Long C Holmes EC Allen RL Wilson J Conlon C Clustered mutations in HIV-1 gag are consistently required for escape from HLA-B27-restricted cytotoxic T lymphocyte responses. J Exp Med (2001) 193:375–86. 10.1084/jem.193.3.375 11157057 PMC2195921

[26] Altfeld M Allen TM Kalife ET Frahm N Addo MM Mothe BR The majority of currently circulating human immunodeficiency virus type 1 clade B viruses fail to prime cytotoxic T-lymphocyte responses against an otherwise immunodominant HLA-A2-restricted epitope: implications for vaccine design. J Virol (2005) 79:5000–5. 10.1128/JVI.79.8.5000-5005.2005 15795285 PMC1069570

[27] Goulder PJ Phillips RE Colbert RA McAdam S Ogg G Nowak MA Late escape from an immunodominant cytotoxic T-lymphocyte response associated with progression to AIDS. Nat Med (1997) 3:212–7. 10.1038/nm0297-212 9018241

[28] Boulter JM Glick M Todorov PT Baston E Sami M Rizkallah P Stable, soluble T-cell receptor molecules for crystallization and therapeutics. Protein Eng (2003) 16:707–11. 10.1093/protein/gzg087 14560057

[29] Laugel B Boulter JM Lissin N Vuidepot A Li Y Gostick E Design of soluble recombinant T cell receptors for antigen targeting and T cell inhibition. J Biol Chem (2005) 280:1882–92. 10.1074/jbc.M409427200 15531581

[30] Garboczi DN Ghosh P Utz U Fan QR Biddison WE Wiley DC. Structure of the complex between human T-cell receptor, viral peptide and HLA-A2. Nature (1996) 384:134–41. 10.1038/384134a0 8906788

[31] Cole DK Dunn SM Sami M Boulter JM Jakobsen BK Sewell AK. T cell receptor engagement of peptide-major histocompatibility complex class I does not modify CD8 binding. Mol Immunol (2008) 45:2700–9. 10.1016/j.molimm.2007.12.009 18243322

[32] Wyer JR Willcox BE Gao GF Gerth UC Davis SJ Bell JI T cell receptor and coreceptor CD8 alphaalpha bind peptide-MHC independently and with distinct kinetics. Immunity (1999) 10:219–25. 10.1016/S1074-7613(00)80022-9 10072074

[33] Gostick E Cole DK Hutchinson SL Wooldridge L Tafuro S Laugel B Functional and biophysical characterization of an HLA-A*6801-restricted HIV-specific T cell receptor. Eur J Immunol (2007) 37:479–86. 10.1002/eji.200636243 17273992 PMC2699040

[34] Cole DK Gallagher K Lemercier B Holland CJ Junaid S Hindley JP Modification of the carboxy-terminal flanking region of a universal influenza epitope alters CD4(+) T-cell repertoire selection. Nat Commun (2012) 3:665 10.1038/ncomms1665 22314361 PMC3293629

[35] Rudolph MG Stanfield RL Wilson IA. How TCRs bind MHCs, peptides, and coreceptors. Annu Rev Immunol (2006) 24:419–66. 10.1146/annurev.immunol.23.021704.115658 16551255

[36] Chesla SE Selvaraj P Zhu C. Measuring two-dimensional receptor-ligand binding kinetics by micropipette. Biophys J (1998) 75:1553–72. 10.1016/S0006-3495(98)74074-3 9726957 PMC1299830

[37] Huang J Edwards LJ Evavold BD Zhu C. Kinetics of MHC-CD8 interaction at the T cell membrane. J Immunol (2007) 179:7653–62. 10.4049/jimmunol.179.11.7653 18025211

[38] Bulek AM Madura F Fuller A Holland CJ Schauenburg AJ Sewell AK TCR/pMHC optimized protein crystallization screen. J Immunol Methods (2012) 382:203–10. 10.1016/j.jim.2012.06.007 22705983 PMC3404460

[39] Winter G Lobley CM Prince SM. Decision making in xia2. Acta Crystallogr D Biol Crystallogr (2013) 69:1260–73. 10.1107/S0907444913015308 23793152 PMC3689529

[40] Collaborative Computational Project, Number 4. The CCP4 suite: programs for protein crystallography. Acta Crystallogr D Biol Crystallogr (1994) 50:760–3. 10.1107/S0907444994003112 15299374

[41] McCoy AJ Grosse-Kunstleve RW Adams PD Winn MD Storoni LC Read RJ. Phaser crystallographic software. J Appl Crystallogr (2007) 40:658–74. 10.1107/S0021889807021206 19461840 PMC2483472

[42] Emsley P Cowtan K. Coot: model-building tools for molecular graphics. Acta Crystallogr D Biol Crystallogr (2004) 60:2126–32. 10.1107/S0907444904019158 15572765

[43] DeLano WL The PyMOL Molecular Graphics System. (2002).

[44] Pace CN Vajdos F Fee L Grimsley G Gray T. How to measure and predict the molar absorption coefficient of a protein. Protein Sci (1995) 4:2411–23. 10.1002/pro.5560041120 8563639 PMC2143013

[45] Fuller A Wall A Crowther MD Lloyd A Zhurov A Sewell AK Thermal stability of heterotrimeric pMHC proteins as determined by circular dichroism spectroscopy. Bio Protoc (2017) 7:e2366. 10.21769/BioProtoc.2366 28748203 PMC5524174

[46] Venyaminov S Baikalov IA Shen ZM Wu CS Yang JT. Circular dichroic analysis of denatured proteins: inclusion of denatured proteins in the reference set. Anal Biochem (1993) 214:17–24. 10.1006/abio.1993.1450 8250221

[47] Tungatt K Bianchi V Crowther MD Powell WE Schauenburg AJ Trimby A Antibody stabilization of peptide-MHC multimers reveals functional T cells bearing extremely low-affinity TCRs. J Immunol (2015) 194:463–74. 10.4049/jimmunol.1401785 25452566 PMC4273996

[48] Lissina A Ladell K Skowera A Clement M Edwards E Seggewiss R Protein kinase inhibitors substantially improve the physical detection of T-cells with peptide-MHC tetramers. J Immunol Methods (2009) 340:11–24. 10.1016/j.jim.2008.09.014 18929568 PMC3052435

[49] Dolton G Tungatt K Lloyd A Bianchi V Theaker SM Trimby A More tricks with tetramers: a practical guide to staining T cells with peptide-MHC multimers. Immunology (2015) 146:11–22. 10.1111/imm.12499 26076649 PMC4552497

[50] Rossjohn J Gras S Miles JJ Turner SJ Godfrey DI McCluskey J. T cell antigen receptor recognition of antigen-presenting molecules. Annu Rev Immunol (2015) 33:169–200. 10.1146/annurev-immunol-032414-112334 25493333

[51] Bridgeman JS Sewell AK Miles JJ Price DA Cole DK Structural and biophysical determinants of alphabeta T-cell antigen recognition. Immunology (2012) 135:9–18. 10.1111/j.1365-2567.2011.03515.x 22044041 PMC3246648

[52] Cole DK Pumphrey NJ Boulter JM Sami M Bell JI Gostick E Human TCR-binding affinity is governed by MHC class restriction. J Immunol (2007) 178:5727–34. 10.4049/jimmunol.178.9.5727 17442956

[53] Miles JJ Bulek AM Cole DK Gostick E Schauenburg AJ Dolton G Genetic and structural basis for selection of a ubiquitous T cell receptor deployed in Epstein-Barr virus infection. PLoS Pathog (2010) 6:e1001198. 10.1371/journal.ppat.1001198 21124993 PMC2987824

[54] Bulek AM Cole DK Skowera A Dolton G Gras S Madura F Structural basis for the killing of human beta cells by CD8(+) T cells in type 1 diabetes. Nat Immunol (2012) 13:283–9. 10.1038/ni.2206 22245737 PMC3378510

[55] Cole DK Bulek AM Dolton G Schauenberg AJ Szomolay B Rittase W Hotspot autoimmune T cell receptor binding underlies pathogen and insulin peptide cross-reactivity. J Clin Invest (2016) 126:3626 10.1172/JCI85679 PMC5004936 27525441

[56] Stepanek O Prabhakar AS Osswald C King CG Bulek A Naeher D Coreceptor scanning by the T cell receptor provides a mechanism for T cell tolerance. Cell (2014) 159:333–45. 10.1016/j.cell.2014.08.042 25284152 PMC4304671

[57] Wooldridge L van den Berg HA Glick M Gostick E Laugel B Hutchinson SL Interaction between the CD8 coreceptor and major histocompatibility complex class I stabilizes T cell receptor-antigen complexes at the cell surface. J Biol Chem (2005) 280:27491–501. 10.1074/jbc.M500555200 15837791 PMC2441837

[58] Laugel B van den Berg HA Gostick E Cole DK Wooldridge L Boulter J Different T cell receptor affinity thresholds and CD8 coreceptor dependence govern cytotoxic T lymphocyte activation and tetramer binding properties. J Biol Chem (2007) 282:23799–810. 10.1074/jbc.M700976200 17540778

[59] Choi EM Chen JL Wooldridge L Salio M Lissina A Lissin N High avidity antigen-specific CTL identified by CD8-independent tetramer staining. J Immunol (2003) 171:5116–23. 10.4049/jimmunol.171.10.5116 14607910

[60] Dolton G Lissina A Skowera A Ladell K Tungatt K Jones E Comparison of peptide-major histocompatibility complex tetramers and dextramers for the identification of antigen-specific T cells. Clin Exp Immunol (2014) 177:47–63. 10.1111/cei.12339 24673376 PMC4089154

[61] Miles KM Miles JJ Madura F Sewell AK Cole DK. Real time detection of peptide-MHC dissociation reveals that improvement of primary MHC-binding residues can have a minimal, or no, effect on stability. Mol Immunol (2011) 48:728–32. 10.1016/j.molimm.2010.11.004 21130497 PMC3032881

[62] Szomolay B Liu J Brown PE Miles JJ Clement M Llewellyn-Lacey S Identification of human viral protein-derived ligands recognized by individual major histocompatibility complex class I (MHCI)-restricted T-cell receptors. Immunol Cell Biol (2016) 94(6):573–82. 10.1038/icb.2016.12 26846725 PMC4943067

[63] Bianchi V Bulek A Fuller A Lloyd A Attaf M Rizkallah PJ Abrogates glycoprotein 100 (gp100) T-cell receptor (TCR) targeting of a human melanoma antigen. J Biol Chem (2016) 291:8951–9. 10.1074/jbc.M115.707414 26917722 PMC4861463

