# Supplementary material for: Dual Molecular Mechanisms Govern Escape at Immunodominant HLA A2-Restricted HIV Epitope
Source: Front Immunol. 2017 Nov 10;8:1503. doi: 10.3389/fimmu.2017.01503 (PMC5701626; doi:10.3389/fimmu.2017.01503)
Supplement: Supplementary file 1 [file image_1.pdf]

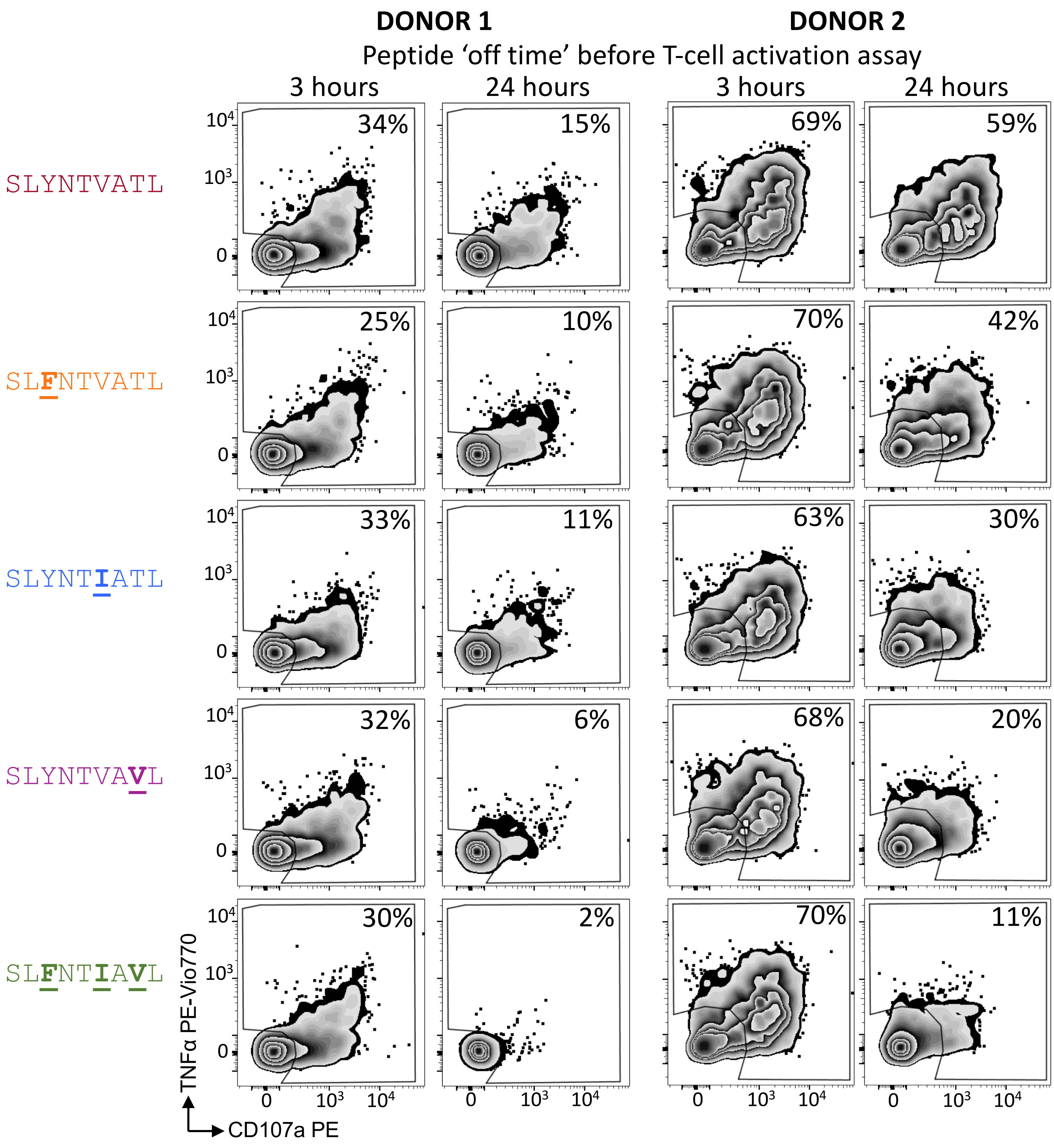

Supplementary Figure 1: **Flow cytometry plots for activation data shown in Figure 8C.** T2 cells were pulsed with peptide for 1 h, washed extensively then allowed to culture for 3 or 24 h before being co-incubated for 5h with T-cells from two donors transduced with the 868 TCR. CD107a and TNFα were used to establish percentage reactivity by flow cytometry. Untransduced CD8+ T-cells did not respond to peptide (data not shown).
